# Effectiveness and Safety of Hormonal Treatments in Children with Growth Disorders: A Systematic Review of Clinical Evidence

**DOI:** 10.3390/clinpract16050096

**Published:** 2026-05-20

**Authors:** Isidro Miguel Martín Pérez, Sebastián Eustaquio Martín Pérez

**Affiliations:** 1 Faculty of Health Sciences, Universidad del Atlántico Medio, Tafira Baja, 35017 Las Palmas, Spain; 2 Hospital Universitario Vithas Las Palmas, Las Palmas de Gran Canaria, 35005 Las Palmas, Spain; 3 Faculty of Health Sciences, Universidad Europea de Canarias, La Orotava, 38300 Santa Cruz de Tenerife, Spain; 4 Faculty of Medicine, Health and Sports, Universidad Europea de Madrid, Villaviciosa de Odón, 28670 Madrid, Spain

**Keywords:** hormone therapy, pubertal disorders, pediatric endocrinology, GnRH, sex steroids, bone age, growth

## Abstract

**Background**: Growth disorders, including central precocious puberty and delayed puberty, can significantly affect linear growth, skeletal maturation, metabolic regulation, and psychosocial development during childhood and adolescence. This systematic review synthesizes the current evidence regarding the effectiveness and safety of hormone-based therapies used in children with disorders of pubertal maturation. **Methods**: A PRISMA-guided systematic search was carried out between January 2016 and March 2026 in different databases, such as MEDLINE (PubMed), EMBASE, CENTRAL, Scopus, Web of Science, CINAHL, LILACS and OpenGrey; the protocol was previously registered in the PROSPERO database (CRD420251068048). Non-randomized, randomized controlled trials and observational research including participants aged 0–18 years receiving hormone therapies were eligible. Risk of bias was assessed using validated, design-specific tools. **Results**: Twenty studies involving 21,812 participants were included. GnRHa therapy improved final adult height (+3.5 to +4.5 cm) and reduced bone age advancement (−0.6 to −1.3 years) in children with central precocious puberty. rhGH therapy increased growth velocity (+3.0 to +5.0 cm/year) and height SDS (+0.3 to +0.9), particularly in idiopathic short stature and Prader–Willi syndrome. Combined GnRHa plus rhGH therapy showed greater short-term growth benefits than GnRHa alone. Both therapies showed favorable safety profiles, with predominantly mild adverse events and discontinuation rates below 2%. However, the evidence was limited by substantial heterogeneity and moderate-to-serious risk of bias. **Conclusions**: GnRHa and rhGH therapies are generally effective and safe for improving growth and pubertal outcomes in pediatric endocrine disorders. However, further long-term studies are needed to clarify their metabolic and psychosocial effects in adulthood. Nevertheless, these conclusions should be interpreted with caution due to the study’s moderate-to-serious risk of bias and heterogeneity.

## 1. Introduction

Puberty is a finely regulated, highly orchestrated and multifactorial biological process defined by the sequential arising of the hypothalamic–pituitary–gonadal (HPG) axis [1,2]. This complex developmental transition culminates in the emergence of secondary sexual characteristics, the attainment of reproductive competence, and profound somatic remodeling, including linear growth acceleration and bone mineral accrual [3]. Although its progression generally follows well-established neuroendocrine pathways [4,5], considerable interindividual and familial variability exists in both the onset and tempo of pubertal maturation. This heterogeneity reflects the interplay of genetic, epigenetic, metabolic and environmental determinants. In evaluating these developmental patterns, it is essential to differentiate between the sequence of pubertal milestones—which is typically conserved— and the timing of their initiation, which is inherently more variable.

When deviations from these expected physiological trajectories occur—manifesting as central precocious puberty (CPP), delayed puberty, or other disorders of pubertal maturation—the consequences extend beyond reproductive maturation [6]. Such disruptions may compromise multiple domains of health during a particularly vulnerable window of physical, endocrine, and psychosocial development [7,8]. Children with altered growth timing frequently exhibit impaired growth potential, discordant bone age (BA) advancement, perturbations in metabolic homeostasis, and suboptimal attainment of peak bone mass [6,9,10].

From a psychosocial perspective, these deviations are often associated with social marginalization, diminished self-concept, heightened emotional reactivity, and increased anxiety symptomatology, collectively exerting a detrimental impact on overall quality of life and academic performance [8,11]. Accordingly, the potential for long-term sequelae underscores the need for early identification, etiological evaluation, and timely, evidence-informed intervention [12]. Importantly, epidemiological evidence suggests that the global age of pubertal onset has progressively declined over recent decades, potentially driven by environmental, nutritional, obesogenic, and endocrine-disrupting exposures. This secular trend has intensified clinical concern regarding both the overdiagnosis and undertreatment of pubertal disorders, further complicating therapeutic decision-making in pediatric endocrinology.

Hormone-based therapies represent the cornerstone of contemporary clinical management for disorders of pubertal maturation and growth-related endocrine conditions [13]. Gonadotropin-releasing hormone analogues (GnRHAs) remain the gold standard for the suppression of CPP, whereas sex steroid replacement strategies (e.g., testosterone, estradiol, and human chorionic gonadotropin) are essential to the induction or support of pubertal progression in cases of delayed puberty or hypogonadotropic states [14,15]. Although these therapies are widely implemented in clinical settings, considerable uncertainties persist concerning the appropriate timing for treatment initiation, optimal therapeutic dosing strategies, and their long-term impact on final adult height (FAH), bone mineral density evolution, cardiometabolic risk profiles, and reproductive outcomes [16,17,18].

Furthermore, although hormone therapies are frequently considered effective in controlling pubertal tempo and improving predicted adult height (PAH), substantial controversy remains regarding the magnitude and durability of these benefits, particularly in relation to long-term skeletal maturation, body composition, fertility potential, and neuropsychological development. Existing studies are often characterized by heterogeneous populations, variable follow-up durations, inconsistent outcome measures, and divergent therapeutic protocols, limiting the comparability and translational applicability of current evidence.

Moreover, heterogeneity in clinical practice across healthcare systems, coupled with the emergence of contemporary longitudinal evidence, underscores the necessity for a rigorous and integrative synthesis of the available data [19,20]. To date, many reviews have focused on isolated endocrine conditions or singular therapeutic approaches, whereas comparatively few studies have comprehensively assessed both efficacy and safety outcomes across the broader spectrum of pediatric disorders of pubertal maturation. Consequently, an important knowledge gap persists regarding the overall benefit–risk balance of hormone therapy during critical stages of growth and developmental plasticity.

Within this context, a comprehensive and rigorous systematic review is warranted to consolidate current evidence and support clinical decision-making. Accordingly, this review aimed to critically evaluate the effectiveness and safety of hormone therapy in pediatric disorders of pubertal maturation. By assessing key outcomes—including pubertal progression, endocrine responses, growth, skeletal maturation, metabolic parameters, and treatment-related safety—this review seeks to provide an updated evidence base to inform clinical management, identify areas of consensus and controversy, and highlight priorities for future research.

## 2. Materials and Methods

### 2.1. Data Sources and Search Strategy

A qualitative systematic review was conducted in accordance with the Preferred Reporting Items for Systematic Reviews and Meta-Analyses (PRISMA) statement [21]. The study protocol was prospectively registered in the PROSPERO database (CRD420251068048; https://www.crd.york.ac.uk/PROSPERO/view/CRD420251068048, retrieved on 25 July 2025), thereby ensuring methodological transparency and adherence to internationally recognized standards for the conduct of systematic reviews.

A literature search was conducted between 1 September 2025 and 31 March 2026 to identify clinical studies assessing the efficacy and safety of hormone therapy in children and adolescents with disorders of pubertal maturation and growth-related endocrine conditions, including precocious puberty, delayed puberty, and central or peripheral disorders of pubertal maturation. The search was performed in the following electronic databases: MEDLINE (PubMed), EMBASE, CENTRAL (Cochrane Library), Scopus, Web of Science (WoS), CINAHL Complete, LILACS, and OpenGrey for grey literature.

The search strategy combined Medical Subject Headings (MeSH) and free-text terms adapted to the syntax of each database to ensure comprehensive coverage. Core terms included “Precocious Puberty” [MeSH], “Delayed Puberty” [MeSH], “Pubertal Disorders” [Title/Abstract], “Pubertal Maturation” [Title/Abstract], “Gonadotropin-Releasing Hormone” [MeSH], “Gonadotropin-Releasing Hormone Agonists” [MeSH], and “Hormone Therapy” [MeSH or Title/Abstract]. Boolean operators were applied systematically.

The primary MEDLINE query was structured as follows: (“Precocious Puberty”[MeSH] OR “Delayed Puberty”[MeSH]) AND (“Hormone Therapy”[MeSH] OR “Gonadotropin-Releasing Hormone”[MeSH]) AND (“Child”[MeSH] OR “Adolescent”[MeSH]). This strategy was supplemented with free-text searches, including combinations such as (“delayed puberty” OR “late puberty” OR “precocious puberty”) AND (“hormone therapy” OR “GnRH agonist”), and (“GnRH agonist*” OR “testosterone replacement” OR “estradiol therapy” OR “hCG therapy”) AND (“puberty disorder*”).

Study selection was conducted in two stages. First, two reviewers independently screened titles and abstracts for eligibility. Second, the full texts of potentially relevant studies were assessed for inclusion. Any discrepancies were resolved through consensus between the reviewers. The complete database-specific search strategies are provided in Supplementary Table S1.

### 2.2. Study Selection

The study selection process followed predefined eligibility criteria to ensure methodological rigor and clinical relevance. Only studies (1) published within the previous 10 years (from 31 January 2016 to 31 March 2026) were considered. Eligible studies included (2) randomized or non-randomized controlled trials, as well as analytical observational studies (e.g., prospective or retrospective cohort studies and case–control studies), (3) involving pediatric population aged 0–18 years (4) diagnosed with disorders of pubertal maturation or growth-related endocrine conditions (e.g., central precocious puberty, delayed puberty, idiopathic short stature, small for gestational age, or related endocrine conditions).

Studies were required to assess (5) hormone-based therapeutic interventions aimed at modulating pubertal progression, skeletal maturation, or auxological outcomes (e.g., GnRHa, sex steroid replacement therapies such as testosterone or estradiol, human chorionic gonadotropin (hCG), or other endocrine treatments). A comparator group was not necessarily mandatory; therefore, single-arm and observational studies without a control were considered when appropriate. When present, comparators included placebo, no treatment, standard care, clinical observation, or alternative hormonal regimens. Additionally, studies had to (6) be available in full-text format (e.g., published or preprint), (7) be written in English, Spanish, or Portuguese, and (8) report at least one clinically relevant outcome, such as pubertal baseline progression, endocrine responses, metabolic parameters, growth, skeletal maturation (e.g., BA), or psychosocial outcomes.

Studies were excluded if they involved (1) adults (>18 years), neonates (0–1 month), or infants under 1 year of age, (2) addressed growth disorders secondary to systemic diseases or iatrogenic conditions, (3) evaluated hormonal therapies unrelated to pubertal maturation or growth-related endocrine management, (4) lacked a relevant endocrine intervention or (5) provided insufficient methodological quality or outcome reporting to allow meaningful interpretation. Moreover, (6) reviews, editorials, conference abstracts without sufficient data, animal studies, in vitro research, and studies without accessible full text were also excluded. Finally, title, abstract, and full-text screening were performed independently by two reviewers (I.M.M.P. and S.E.M.P.). Discrepancies were resolved through consensus between the two reviewers.

### 2.3. Data Extraction

Two authors independently extracted data using a standardized PICO-based template specifically adapted to the clinical characteristics of disorders of pubertal maturation and growth-related endocrine conditions. Extracted variables included: study characteristics (e.g., authors, year and country), study design and population diagnostic criteria. Participant data included sample size, age, sex distribution, baseline Tanner stage and hormonal profile. Regarding interventions, we recorded the type, dosage and treatment duration, along with comparator details (e.g., placebo, no treatment, usual care, or alternative hormonal regimen).

Primary outcomes encompassed pubertal progression, auxological parameters (e.g., height, predicted adult height), BA, hormonal and metabolic changes, psychological well-being, and treatment-related adverse events (AEs). Finally, statistical findings, effect measures, follow-up duration, and authors’ conclusions were documented. To ensure consistency between reviewers, a pilot extraction was conducted following the Cochrane Handbook for Systematic Reviews of Interventions (v.6.4.) [22]. Any discrepancies between evaluators were resolved by consensus or, when necessary, with the assistance of a third reviewer.

### 2.4. Methodological Quality Assessment

The methodological quality of the included studies was assessed using validated and design-specific tools. Randomized controlled trials were evaluated using the Cochrane Risk of Bias tool version 2 (RoB 2) [23], which examines potential bias arising from the randomization process, deviation from intended interventions, missing outcomes data, measurement of the outcomes, and selection of the reported result.

Non-randomized and observational cohort studies were assessed using ROBINS-I (Risk of Bias in Non-randomized Studies of Exposures) tool [24], which is specifically designed to evaluate the risk of bias in studies assessing the effects of interventions without randomization. This tool addresses bias due to confounding, participant selection, classification of interventions, deviations from intended interventions, missing data, measurement of outcomes, and selection of the reported results.

Case series were appraised with the Joanna Briggs Institute (JBI) Critical Appraisal Checklist for Case Studies [25], which evaluates methodological quality in terms of inclusion criteria, condition measurement, completeness of inclusion, reporting of outcomes, follow-up adequacy, and statistical analysis. All quality assessments were conducted independently by two reviewers (I.M.M.P. and S.E.M.P.), and any disagreements were resolved through discussion and consensus.

### 2.5. Data Synthesis

A qualitative synthesis was conducted to summarize and interpret the findings of the included studies. A quantitative meta-analysis was not performed due to substantial clinical and methodological heterogeneity, including differences in study design, participant characteristics, endocrine diagnoses, intervention protocols, follow-up duration, and outcome measures.

Accordingly, findings were synthesized narratively and organized by endocrine condition and hormone-based intervention. Additionally, key outcomes—including pubertal progression, growth, skeletal maturation, hormonal and metabolic responses, psychosocial variables, and treatment safety—were interpreted considering the methodological quality and risk of bias of the included studies.

## 3. Results

### 3.1. Study Selection

The systematic search yielded a total of 162 records across the predefined electronic databases: MEDLINE (PubMed) (n = 34), EMBASE (n = 36), CENTRAL (Cochrane Library) (n = 18), Scopus (n = 27), Web of Science (WoS) (n = 22), CINAHL Complete (n = 15), LILACS (n = 6), and OpenGrey (n = 4). After removal of 29 duplicate entries, 133 unique records proceeded to title and abstract screening.

During the initial screening phase, 87 records were excluded for failing to meet predefined eligibility criteria. The principal reasons for exclusion included ineligible populations (e.g., absence of disorders of pubertal maturation or growth-related endocrine conditions), interventions outside the scope of hormone-based therapies of interest, inappropriate study designs (e.g., case reports, narrative reviews, or conference abstracts without sufficient data), or lack of clinically relevant auxological or endocrine outcomes.

**Figure 1:**
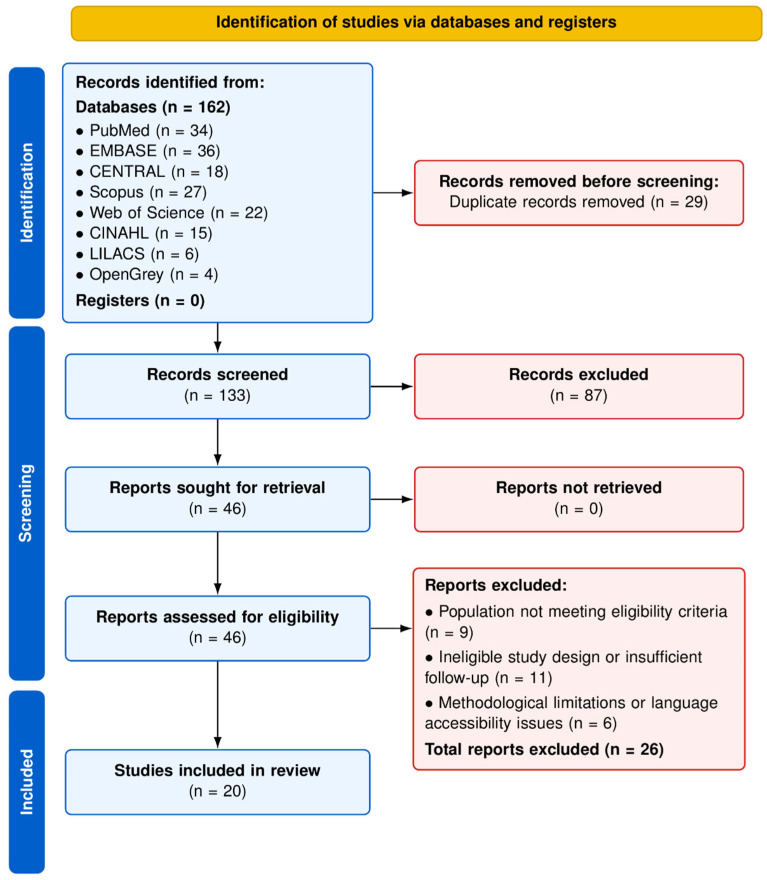
PRISMA 2020 flow diagram. Source: Own work.

A total of 46 full-text articles were subsequently assessed for eligibility. Following a comprehensive evaluation, 26 studies were excluded. The main reasons for exclusion were: (1) populations not fulfilling strict diagnostic criteria for CPP, ISS, SGA, or PWS (n = 9); (2) methodological limitations inconsistent with inclusion standards, including inadequate follow-up duration or absence of quantitative outcome reporting (n = 11); and (3) insufficient methodological transparency or lack of language accessibility precluding full critical appraisal (n = 6). Ultimately, 20 studies satisfied all eligibility criteria and were included in the qualitative synthesis. Figure 1 illustrates the study selection procedure in accordance with the PRISMA 2020 flow diagram.

### 3.2. Characteristics of the Included Studies

Twenty studies [26,27,28,29,30,31,32,33,34,35,36,37,38,39,40,41,42,43,44,45], involving 21,812 children, were included in the qualitative synthesis. The body of evidence comprised RCTs, phase III open-label trials, prospective and retrospective cohort studies, case–control investigations, case series, and large-scale registry-based real-world analyses. The largest contribution originated from a phase IV post-marketing registry evaluating biosimilar rhGH, accounting for 20,465 participants [31], whereas the smallest dataset corresponded to a familial case series of four patients with *MKRN3*-related CPP [29].

With respect to design, four investigations employed randomized methodologies. Kariola et al. evaluated puberty-promoting therapy in boys with CDGP using a randomized, controlled, open-label design [26]. Two phase III randomized trials examined rhGH therapy in prepubertal children with ISS, adopting delayed-treatment parallel-group designs [41,42]. A further randomized, active-controlled, parallel-group phase III trial assessed rhGH in children with PWS, directly comparing two commercially available growth hormone formulations over 52 weeks [40].

Several studies focused on GnRHa therapy in children with CPP. These included retrospective cohorts assessing adult height, BMI trajectories, skeletal maturation, and long-term outcomes in girls and boys with idiopathic CPP [27,28,30,32,34,35], as well as phase III open-label trials evaluating long-acting leuprolide or triptorelin formulations [33,39,43,44,45]. Etiology-specific cohorts were represented, including *MKRN3*-related familial CPP [29] and CPP secondary to hypothalamic hamartoma [37].

Most observational studies relied on within-subject baseline comparisons, reflecting the ethical and practical constraints of pediatric endocrine research, where withholding active therapy may be inappropriate [27,28,29,30,32,33,35,37,39,43,44,45]. The populations were clinically heterogeneous, including girls with idiopathic CPP, boys with idiopathic CPP, children with ISS, infants and children with PWS, patients with GHD, and boys with CDGP.

Sex distribution reflected the epidemiology of the underlying conditions. Most CPP cohorts were predominantly or exclusively female [27,28,34,35], whereas Ünsal et al. specifically evaluated boys with idiopathic CPP [32], and Kariola et al. included only boys with CDGP [26]. Mixed-sex populations predominated in ISS, PWS, registry-based GH studies, and phase III GnRHa trials [31,39,40,41,42,43,44,45].

Therapeutic regimens varied according to diagnosis. GnRHa was administered mainly as depot formulations of leuprolide acetate or triptorelin, commonly at 3.75 mg every 28 days, although longer-acting formulations included 3-month and 6-month preparations [27,28,29,30,32,33,34,35,37,39,43,44,45]. rhGH was administered by daily or near-daily subcutaneous injection in ISS, PWS, GHD, and SGA-related cohorts, using weight-adjusted dosing or titration according to clinical response and IGF-1 monitoring [31,38,40,41,42]. One study compared GnRHa monotherapy with combined GnRHa plus rhGH therapy in girls with CPP and poor height prognosis [34].

Primary outcomes included height SDS, height velocity, PAH, FAH, growth velocity, skeletal maturation indices, and biochemical suppression of the HPG axis [27,29,30,32,33,34,35,39,43,44,45]. Several studies also assessed BMI or BMI-SDS trajectories, showing heterogeneous changes according to sex, baseline adiposity, and treatment duration [27,28,30,32]. Specialized outcomes included temperament and psychosocial parameters in CDGP [26], ocular growth and myopia progression in CPP [36], uterine and ovarian morphology in SGA girls receiving GH [38], and body composition and neurodevelopment in infants with PWS [40].

Safety outcomes were consistently reported across studies and included serious AEs, injection-site reactions, treatment discontinuation, and metabolic or skeletal safety. Overall, GnRHa and rhGH showed favorable safety profiles, with most AEs described as mild or transient [31,39,40,41,42]. Nevertheless, several authors emphasized the need for longitudinal monitoring of BMI, metabolic parameters, skeletal maturation, and recovery during prolonged endocrine therapy [27,28,30,32,38].

Geographically, the evidence base was internationally distributed, including studies from Finland [26], Turkey [27,32], Italy [28], China [29,33,35], Iran [30,31], South Korea [34,36,37,40,41,42], Poland [38], the United States [39,44], and multicenter collaborations across the Americas and Europe [43,45]. Comprehensive methodological and clinical characteristics of all included studies are detailed in Table S2.

### 3.3. Quality Assessment

#### 3.3.1. Randomized Controlled Trials

RCTs, including phase III trials evaluating rhGH in ISS and PWS, as well as trials assessing GnRH analog therapies, were determined with the RoB 2 tool [23]. The included RCTs were judged to present a low to moderate risk of bias. The randomization process (D1) was adequately described, with a sequence generation and balanced baseline characteristics between intervention groups.

Bias due to missing outcome data (D3) and selective reporting (D5) was considered low across most studies, as attrition rates were limited and key outcomes —particularly growth-related variables—were consistently reported. Some concerns were identified in deviations from intended interventions (D2), mainly due to the open-label design common to these trials. However, given that primary outcomes such as height velocity (HV) and height standard deviation score (height SDS) are objective, standardized, and protocol-driven measures, the potential impact of performance bias was considered limited.

Bias in outcome measurement (D4) was generally low, as outcomes were assessed using validated and objective clinical measures. Particular attention was given to the study by Lee et al. (2016) [44], which involved a post hoc analysis; therefore, a higher risk of bias related to selective reporting and analytical flexibility cannot be excluded. A detailed domain-level assessment is presented in Table 1.

#### 3.3.2. Non-Randomized Interventional and Observational Cohort Studies

ROBINS-I tool [24] was applied to non-randomized interventional studies, retrospective cohorts, registry-based investigations, single-arm trials, and observational studies evaluating hormonal therapies such as GnRHa, rhGH, and combined GnRHa plus rhGH interventions [27,28,30,31,32,33,34,35,36,37,38,39,43,45]. Most of the included studies were judged to have a moderate risk of bias, mainly due to confounding, non-randomized treatment allocation, absence of untreated control groups, and retrospective or single-arm designs.

Confounding (D1) was the main source of bias, largely reflecting confounding by indication. Treatment decisions—including initiation, timing, dose adjustments, and combination therapy—were driven by baseline pubertal status, BA advancement, PAH, or growth prognosis [27,28,30,32,33,34,35,37,38,39,43,45]. Furthermore, bias in participant selection (D2) and missing data (D5) was generally low to moderate, particularly in retrospective and long-term studies where inclusion depended on complete follow-up or availability of FAH data [27,28,30,32,34,35,37,38]. Single-arm and registry-based studies also lacked appropriate comparators or complete longitudinal data [31,33,39,43].

Bias in classification of interventions (D3) and outcome measurement (D6) was consistently low, as treatments were clearly defined and outcomes were objective and standardized, including height SDS, BA, PAH or FAH, and hormonal markers [27,28,30,31,32,33,34,35,37,38,39,43,45]. Bias due to deviations from intended interventions (D4) was rated as low to moderate, reflecting real-world treatment modifications such as dose adjustments or addition of rhGH [27,30,34,35]. Bias in selection of the reported result (D7) was generally low, although some concerns remained in retrospective and post hoc analyses [34,38,44,45]. Overall, the evidence was considered to have a moderate risk of bias, with greater concern in studies with small sample sizes, single-arm designs, or incomplete long-term follow-up. Detailed domain-level assessments are presented in Table 2.

#### 3.3.3. Case Series

The study by Chen et al. [29] was classified as a case series and appraised using the JBI Critical Appraisal Checklist for Case Series. Eligibility criteria for familial CPP with genetically confirmed *MKRN3* mutations were defined, and diagnosis was based on standardized clinical, hormonal and molecular assessments. Participant characteristics and relevant auxological data were adequately reported.

However, consecutive case inclusion was not specified, and the small sample size (n = 4) limited representativeness. In addition, follow-up was restricted to short- and medium-term outcomes, with no data on FAH. Although several JBI domains were fulfilled, limitations related to sample size, potential selection bias, and limited follow-up justified classification as high risk of bias. Nevertheless, the study provides relevant descriptive evidence in a rare monogenic form of CPP. A detailed appraisal is presented in Table 3.

### 3.4. Main Results

#### 3.4.1. Effectiveness Outcomes

- *Growth Outcomes and Final Adult Height*

Auxological outcomes were the most frequently reported endpoints and included height SDS/HSDS, growth velocity, PAH and FAH. Overall, treatment effects varied according to the underlying endocrine condition, baseline growth potential, timing of treatment, and therapeutic strategy.

In girls with idiopathic CPP, GnRHa therapy was consistently associated with modest but clinically relevant improvements in adult height prognosis. For example, Akın and Özgen [27] reported gains of approximately 4 cm in FAH compared with pretreatment PAH (*p* < 0.001), whereas Maleki et al. [30] observed FAH values exceeding target height following long-term treatment.

Among patients with poor height prognosis, combined GnRHa plus rhGH therapy showed greater short-term growth benefits than GnRHa monotherapy, particularly regarding growth velocity and PAH [34,35]. Cho et al. [34] reported greater height gain in the combined therapy group (9.2 vs. 4.7 cm, *p* < 0.001), although differences in FAH remained non-significant.

In boys with idiopathic CPP and hypothalamic hamartoma–associated CPP, GnRHa therapy generally preserved growth potential and improved PAH [32,37]. However, growth responses appeared more limited in *MKRN3*-related familial CPP, with only modest changes in height SDS and PAH during treatment [29].

Higher-quality evidence from controlled studies in ISS showed that rhGH therapy significantly accelerated linear growth. Chung et al. [41] reported an increase in annual growth velocity from 5.6 to 10.1 cm/year after treatment (*p* < 0.001), while Kim et al. [42] observed significantly greater gains in height SDS compared with untreated controls.

In PWS, rhGH therapy produced sustained improvements in height SDS and body composition [38,40]. In boys with constitutional delay of growth and puberty (CDGP), puberty-promoting therapy did not negatively affect psychosocial well-being, while letrozole improved sociability compared with testosterone [26].

- *Skeletal Maturation and Bone Age Advancement*

Across studies involving CPP, GnRHa therapy was consistently associated with slower skeletal maturation, reflected by reductions in BA advancement relative to CA [30,33,39,43,44,45]. For instance, Maleki et al. [30] reported a significant reduction in the BA–CA difference, from 1.5 to 0.7 years (*p* < 0.001), supporting the role of GnRHa in slowing skeletal progression. Comparable findings were also observed across monthly, 3-month, and 6-month GnRHa formulations, supporting a consistent therapeutic effect on skeletal progression [39,43,44,45].

In hypothalamic hamartoma–associated CPP, skeletal advancement also decreased during treatment [37], whereas responses appeared less pronounced in *MKRN3*-related familial CPP [29]. In contrast, in children with ISS and PWS receiving rhGH therapy, skeletal maturation generally progressed proportionally to CA, without evidence of pathological acceleration [38,40,41,42].

- *Body Mass Index and Anthropometric Composition*

BMI trajectories varied according to diagnosis and baseline adiposity. In girls with idiopathic CPP, GnRHa therapy was associated with modest increases in BMI-SDS during treatment, although these changes frequently stabilized or improved after treatment discontinuation [27,28,30]. For example, Cammisa et al. [28] indicated an increase in BMI-for-age z-score from 0.5 at baseline to 0.8 after one year of treatment (*p* = 0.015), without significant changes in obesity prevalence.

In boys with idiopathic CPP, BMI responses differed according to baseline weight status. Ünsal et al. [32] observed reductions in BMI-SDS among boys with obesity during treatment, whereas normal-weight children showed only transient increases. In ISS, rhGH therapy did not produce clinically relevant changes in BMI-SDS [41,42].

In contrast, children with PWS showed significant improvements in body composition during rhGH therapy, including increased lean body mass and reduced body fat percentage. In this sense, Yang et al. [40] reported reductions in body fat of approximately 7–8% during follow-up, suggesting metabolic benefits beyond linear growth. Interestingly, Chen et al. [29] reported reductions in BMI-SDS and obesity prevalence in children with *MKRN3*-related familial CPP during GnRHa therapy.

- *Endocrine, Hormonal, and Psychocognitive Outcomes*

GnRHa therapy consistently achieved effective suppression of the HPG axis across studies. High rates of hormonal suppression were reported, with Yu et al. [33] observing LH suppression exceeding 98% after 12 months of triptorelin therapy. Comparable endocrine control was observed across different GnRHa formulations, supporting their effectiveness in regulating pubertal progression [43,44,45].

In populations receiving rhGH therapy, IGF-1 and IGFBP-3 levels increased during treatment while remaining within expected physiological and safety ranges [38,40,41,42], indicating an appropriate endocrine response to therapy. Psychocognitive outcomes were evaluated less frequently; however, available evidence suggested potential benefits in emotional regulation, sociability, motor function, and cognitive development in selected pediatric populations [26,40].

#### 3.4.2. Safety Outcomes

GnRHa and rhGH therapies showed favorable safety profiles across studies. Most reported AEs were mild and transient, commonly including headache, nasopharyngitis, pyrexia, injection-site pain, and upper respiratory tract infections [31,39,40,41,42,43].

Evidence from large observational cohorts further supported treatment safety. In the Orchid-Life registry, serious AEs occurred in only 0.08% of patients, and treatment discontinuation due to AEs was rare [31]. Similarly, long-acting GnRHa formulations did not show major treatment-related safety concerns, and no clinically relevant AEs on bone maturation, glucose metabolism, thyroid function, or overall endocrine health were reported during follow-up [27,38,40,41,42].

**Figure 2:**
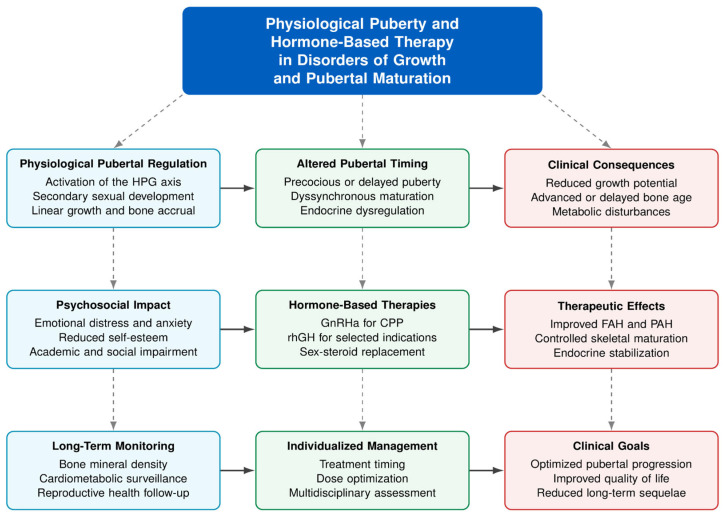
Integrated conceptual framework of the effectiveness and safety of GnRHa and rhGH therapies in pediatric endocrine disorders. Blue boxes represent physiological puberty regulation, green boxes endocrine dysregulation and treatment strategies, and red boxes clinical outcomes and long-term goals. Arrows show relationships between pubertal development, therapeutic interventions, and expected outcomes. Abbreviations: HPG, hypothalamic–pituitary–gonadal; GnRHa, gonadotropin-releasing hormone agonists; CPP, central precocious puberty; rhGH, recombinant human growth hormone; FAH, final adult height; PAH, predicted adult height. Source: Own work.

Nevertheless, several studies emphasized the importance of monitoring BMI trajectories and metabolic parameters during prolonged treatment, particularly in children with baseline overweight or obesity [27,30,32]. A concise summary of the main findings is presented in Table S3 and Figure 2.

## 4. Discussion

The present synthesis indicates that the therapeutic effects of GnRHa and rhGH are likely influenced by the interaction of multiple interacting factors, including diagnosis, baseline auxological profile, pubertal stage at treatment initiation, treatment duration and therapeutic sequencing. Nevertheless, interpretation of effectiveness should be contextualized according to methodological quality if available evidence, as most included studies were observational and judged to have a moderate risk of bias.

In this context, evidence regarding GnRHa therapy in CPP was derived predominantly from retrospective and non-randomized studies, which consistently suggested beneficial effects on pubertal control and slowing of skeletal maturation. Conversely, evidence supporting short-term improvements in HV and height SDS with rhGH in ISS and PWS was more consistently supported by randomized and controlled trial data [27,30,34,35,37,38,39,40,41,42].

Contemporary cohort studies, meta-analyses, and consensus recommendations further suggest that treatment response in CPP is highly dependent on pubertal phenotype, baseline BA advancement, and timing of intervention [46,47,48,49,50]. Corripio et al. reported favorable FAH outcomes in girls with idiopathic CPP treated with triptorelin, while a meta-analysis by Chen et al. suggested that clinically meaningful height benefits may still be achieved in girls older than six years receiving GnRHa therapy [46,47]. Similarly, Saito and Hasegawa emphasized that adult height outcomes remain heterogeneous and appear strongly influenced by treatment timing and baseline growth potential [48].

These findings are consistent with retrospective evidence included in the present review, suggesting that GnRHa initiation around eight years of age may still be associated with modest improvements in FAH in selected patients [27,49]. However, the magnitude of benefit remains variable across studies, and interpretation should acknowledge the predominance of observational evidence, heterogeneity in treatment protocols, and the limited availability of long-term controlled comparisons. Accordingly, therapeutic decision-making should remain individualized and guided by baseline auxological characteristics, skeletal maturation status, and expected height prognosis.

### 4.1. Effectiveness of Hormone Therapy on Pubertal Control and Growth

In idiopathic CPP, premature activation of the HPG axis accelerates skeletal maturation and epiphyseal fusion through early estrogen exposure at the growth plate. Experimental and translational evidence indicates that estrogens contribute to the progressive depletion of resting-zone progenitor cells, thereby shortening the period available for longitudinal growth and ultimately compromising FAH potential [51,52,53]. Within this biological framework, GnRHa therapy seeks to suppress premature reactivation of the HPG axis by inhibiting gonadotropin secretion and reducing estrogen-mediated skeletal progression, thus slowing skeletal advancement and delaying epiphyseal maturation [27,30,33,34].

This biological rationale provides the theoretical basis for GnRHa treatment in CPP, whose clinical effectiveness has been evaluated predominantly through retrospective and observational designs. Meta-analyses and cohort investigations suggest that GnRHa treatment may confer modest yet clinically meaningful benefits in FAH, particularly when initiated before marked advancement of skeletal maturation occurs [46,47,48]. Nevertheless, these findings should be interpreted cautiously due to heterogeneity in treatment timing, baseline auxological characteristics, duration of follow-up, and methodological quality across studies.

Within the studies included in this review, Akın and Özgen reported an average FAH gain of approximately 4 cm relative to PAH among girls with idiopathic CPP, although the observational nature of the study and absence of untreated controls limit causal interpretation [27]. Similarly, Yang et al. observed that pharmacological treatment in CPP and early puberty was associated with improved pubertal control and selected auxological parameters, although therapeutic response varied substantially according to baseline growth potential and pubertal phenotype [50].

Combined therapy with GnRHa and rhGH has emerged as a potential strategy for patients presenting with poor height prognosis or pronounced growth deceleration during pubertal suppression. Recent systematic reviews and meta-analyses indicate that combined treatment may produce greater improvements in HV and PAH than GnRHa monotherapy; however, evidence regarding superior FAH outcomes remains inconsistent [51]. In the retrospective studies included in this review, Cho et al. and Shi et al. reported greater short-term gains in HV, height SDS, and PAH among children receiving combined therapy [34,35]. However, interpretation should remain cautious, as treatment allocation was likely influenced by baseline prognosis, thereby introducing potential selection bias and confounding by indication.

Earlier findings by Wang et al. similarly suggested enhanced auxological outcomes among Chinese children with CPP treated with combined GnRHa and rhGH therapy [52]. Consequently, while combined treatment may offer benefits in carefully selected patients—particularly those with markedly compromised PAH or low baseline height SDS—clinical decisions should remain personalized and guided by the risk–benefit considerations. The rationale supporting rhGH therapy is grounded in the anabolic actions of the GH–IGF-1 axis on chondrocyte proliferation, extracellular matrix synthesis, and longitudinal bone growth [53]. Contemporary evidence further suggests that GH, GnRHa, and aromatase inhibitors may act synergistically in selected disorders affecting pubertal maturation and somatic growth through modulation of skeletal maturation and extension of growth plate activity [54].

Among children with ISS, the strongest evidence originated from RCTs and systematic reviews, which consistently showed short-term improvements in HV and height SDS following rhGH therapy [41,42,55]. However, uncertainty persists regarding the long-term durability of these gains and their translation into clinically meaningful improvements in FAH. Chen et al. similarly reported favorable growth responses among school-aged children with ISS treated with rhGH, while emphasizing the importance of individualized treatment expectations and careful interpretation of adult height outcomes [56]. Emerging evidence also suggests that aromatase inhibition may represent a complementary strategy for prolonging growth plate activity during adolescence. In this regard, Cui et al. showed that combined aromatase inhibitor and GH therapy improved auxological parameters in male adolescents with ISS, reinforcing the role of skeletal maturation control in optimizing adult height potential [57].

In PWS, the benefits of rhGH therapy appear to extend beyond linear growth. Current international recommendations advocate early treatment initiation within multidisciplinary care pathways because of the broad metabolic, functional, and developmental benefits associated with GH therapy [58,59,60]. Observational evidence suggests that early rhGH treatment may attenuate progression toward severe obesity, improve body composition, and increase lean body mass (LBM) [61,62,63]. Moreover, long-term studies have reported potential benefits in motor development, bone mineralization, and quality of life throughout childhood and adulthood, although much of the evidence remains observational and should therefore be interpreted accordingly [62,63].

### 4.2. Effectiveness on Skeletal Maturation and Bone Health

Attenuation of BA advancement was one of the most consistently reported findings associated with GnRHa therapy across studies involving CPP. Observational cohorts and phase III investigations generally reported reductions in the BA–CA gap, suggesting a beneficial effect on skeletal maturation and preservation of growth potential [27,30,33,37,39,43,44,45]. Nevertheless, interpretation of treatment magnitude should remain cautious, as the available evidence is derived predominantly from retrospective cohorts, single-arm studies, and non-randomized designs, thereby limiting causal inference regarding long-term skeletal outcomes.

From a mechanistic perspective, estrogens play a pivotal role in growth plate senescence and epiphyseal fusion, providing a strong biological rationale for GnRHa therapy in CPP [56,58]. Experimental evidence suggests that estrogen-mediated depletion of resting-zone progenitor cells contributes to accelerated skeletal maturation and premature closure of growth plates [63]. Accordingly, suppression of the HPG axis through GnRHa therapy may delay skeletal progression by reducing estrogen exposure during critical periods of growth [27,30,33,34].

Evidence from phase III and observational studies further suggests that long-acting GnRHa formulations, including triptorelin and leuprolide depot preparations, may achieve comparable endocrine suppression and control of skeletal maturation, although direct head-to-head randomized comparisons remain scarce [33,39,43,44,45]. Across studies included in this review, these formulations were consistently associated with stabilization or regression of pubertal progression together with reductions in the BA–CA discrepancy, supporting their clinical utility in preserving adult height potential. In line with these findings, current international consensus statements support GnRHa as the standard therapeutic approach for progressive CPP, particularly when accelerated skeletal maturation threatens FAH [61].

In specific CPP subgroups, treatment responses appeared more heterogeneous. In children with CPP secondary to hypothalamic hamartoma, GnRHa therapy was associated with improvements in skeletal maturation and PAH, supporting its effectiveness in this neuroendocrine context [37]. Conversely, in familial CPP associated with *MKRN3*-related mutations, BA progression and auxological outcomes appeared comparatively less responsive despite adequate pubertal suppression, suggesting that genetic determinants regulating pubertal timing may also influence downstream skeletal dynamics and treatment responsiveness [29].

The physiology of growth plate closure has received increasing scientific attention in recent years. Cho et al. highlighted the interaction between estrogen signaling, chondrocyte senescence, and epiphyseal fusion as a key determinant of skeletal maturation [56]. Similarly, experimental findings by Nilsson et al. indicated that estrogens accelerate growth plate senescence through progressive depletion of progenitor cell populations [64]. Collectively, these mechanistic findings provide robust biological plausibility supporting the clinical rationale for GnRHa therapy in CPP.

In contrast to CPP, evidence from RCTs and observational studies involving ISS and PWS suggests that rhGH therapy does not induce pathological acceleration of skeletal maturation when administered under appropriate monitoring [38,40,41,42]. Across these populations, BA progression generally remained proportional to CA, indicating preservation of skeletal development. Although long-term rhGH treatment appears compatible with normal skeletal maturation, monitoring of BA progression remains advisable in practice to optimize treatment timing and avoid excessive advancement [59]. In PWS specifically, rhGH therapy may additionally contribute to improved bone mineral density and skeletal metabolism, although alterations in bone turnover markers relative to healthy populations have been reported, warranting continued longitudinal surveillance [64].

### 4.3. Safety and Tolerability

GnRHa and rhGH therapies showed favorable safety and tolerability profiles across the included studies. However, interpretation of safety outcomes should be contextualized according to the methodological quality of the available evidence, as long-term safety data were derived predominantly from observational cohorts, registry-based investigations, and open-label studies, with comparatively limited randomized evidence. Across included studies, most reported AEs were mild and transient, commonly including injection-site reactions, headache, nasopharyngitis, pyrexia, and upper respiratory tract infections [31,39,40,41,42,43,44,45]. Large post-marketing registries and observational surveillance programs have also supported the overall safety of pediatric GH therapy when administered according to established clinical indications and monitoring protocols [65,66].

In children receiving GnRHa therapy, metabolic changes should be interpreted within the context of physiological endocrine adaptation accompanying pubertal suppression. Several observational studies reported modest and transient increases in BMI-SDS, particularly among girls with idiopathic CPP [27,28,30]. Importantly, these changes appeared to be influenced by baseline anthropometric status. In this regard, Bruzzi et al. indicated that pre-treatment BMI significantly modulates long-term metabolic and auxological trajectories during GnRHa therapy, highlighting the importance of individualized metabolic monitoring [65]. Nevertheless, most studies included in this review suggested that obesity prevalence does not increase substantially during treatment and that BMI values frequently stabilize or normalize following treatment discontinuation [27,28].

Among boys with CPP, BMI trajectories appeared more heterogeneous and highly dependent on baseline weight status [32]. In particular, obese children showed reductions in BMI-SDS during treatment, whereas normal-weight individuals frequently exhibited transient increases without clinically relevant long-term deterioration. These findings reinforce the relevance of nutritional counseling, lifestyle modification, and longitudinal metabolic surveillance in routine endocrine management, particularly during prolonged pubertal suppression.

The safety profile of rhGH therapy appeared similarly reassuring across pediatric endocrine conditions. Evidence from long-term observational registries—including the NordiNet International Outcome Study (NordiNet IOS) [67], the American Norditropin Studies: Web-Enabled Research (ANSWER) Program [68], the PATRO Children study [69], and the Genetics and Neuroendocrinology of Short Stature International Study (GeNeSIS) [70]—reported favorable tolerability without consistent evidence of increased serious treatment-related complications. In disorders such as ISS and Turner syndrome, rhGH therapy was generally associated with mild AEs and no clear signal of clinically relevant metabolic toxicity, although continued monitoring remains essential during long-term administration.

In PWS, careful clinical monitoring is particularly important due to the high prevalence of comorbidities throughout development [71]. Nevertheless, evidence suggests that rhGH therapy may improve body composition, metabolic function, and quality of life when administered within structured programs [59,63,64]. In this context, longitudinal monitoring of respiratory function, glucose metabolism, thyroid status, and sleep-disordered breathing remains especially relevant to optimize safety and treatment effectiveness [59,60,71].

Endocrine monitoring across studies further supported the biological effectiveness and safety of hormone therapy. In children receiving GnRHa, sustained suppression of LH, FSH, estradiol, and testosterone concentrations was consistently reported, together with stabilization or regression of secondary sexual characteristics [29,33,39,43,44,45]. Likewise, rhGH therapy was associated with expected increases in IGF-1 and IGFBP-3, generally remaining within recommended therapeutic safety ranges [31,38,40,41,42]. To sum up, these findings support a favorable short- and medium-term safety profile for both GnRHa and rhGH therapies, although the predominance of observational evidence highlights the need for additional long-term randomized and prospective safety studies.

### 4.4. Limitations

Several limitations should be considered when interpreting the findings of this review. First, the scope of the included literature was highly heterogeneous with respect to study design, patient populations, endocrine conditions, therapeutic protocols, outcome definitions, and follow-up duration. Such variability limits direct comparability between studies and complicates the interpretation of pooled clinical conclusions.

In particular, differences in treatment initiation age, pubertal stage, dosage regimens, and duration of GnRHa or rhGH therapy may explain the inconsistencies observed not only in FAH but also in bone maturation and metabolic outcomes. Additionally, the substantial methodological and clinical heterogeneity across studies precluded the performance of a formal meta-analysis, limiting the possibility of quantitatively synthesizing effect sizes and generating robust pooled estimates.

Second, the predominance of observational and non-randomized studies increases the risk of selection bias, residual confounding, and uncontrolled maturation-related effects. Many studies relied primarily on within-subject baseline comparisons rather than parallel external control groups, which reduces the strength of causal inference and may overestimate treatment-related effects due to normal developmental progression and secular growth trends.

Third, our results indicated limitations regarding the long-term comparability of outcomes. Measures such as PAH, BA advancement, and BMI-SDS, although clinically relevant, represent surrogate markers rather than definitive indicators of long-term metabolic, functional, or psychosocial health. Moreover, follow-ups were frequently insufficient to assess sustained adult outcomes, particularly regarding cardiovascular risk, body composition, fertility, psychological well-being, and quality of life in adulthood.

Finally, psychosocial and patient-reported outcomes were inconsistently evaluated across studies despite their recognized relevance in pediatric endocrine disorders. The limited assessment of emotional well-being, social functioning, self-perception, and treatment burden represents an important gap in the current literature [72,73].

### 4.5. Implications for Clinical Practice

From a clinical perspective, the available evidence supports the cautious use of hormone-based therapies within defined clinical indications and under specialist supervision. However, recommendations should be interpreted proportionally to the quality of the underlying evidence, which remains predominantly observational for several endocrine conditions.

**Figure 3:**
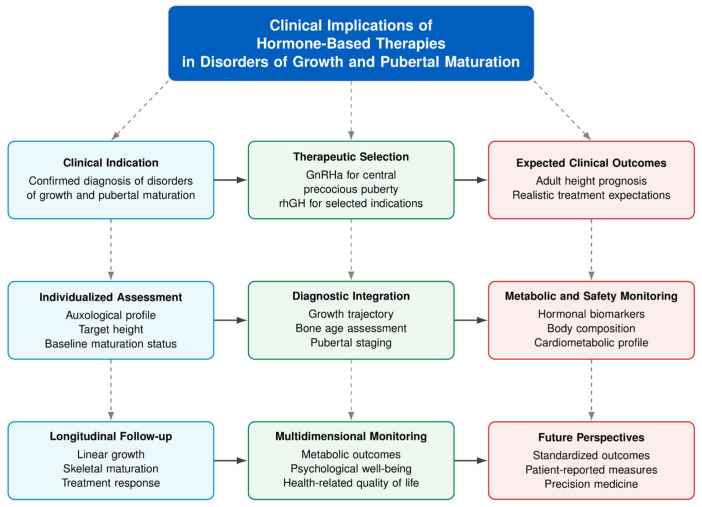
Summary of the main implications for clinical practice. Hormone-based therapies should be prescribed according to clinical indications, individualized assessment, specialist supervision, and longitudinal multidimensional monitoring. Blue boxes represent clinical indication, individualized assessment, and longitudinal follow-up. Green boxes illustrate therapeutic selection, diagnostic integration, and multidimensional monitoring. Red boxes summarize expected outcomes, safety monitoring, and future perspectives. Arrows indicate the relationships between diagnosis, treatment strategies, monitoring, and long-term clinical goals. Abbreviations: GnRHa, gonadotropin-releasing hormone agonists; rhGH, recombinant human growth hormone. Source: Own work.

GnRHa continues to represent the standard therapeutic strategy for CPP and other conditions characterized by premature activation of the HPG axis. In contrast, rhGH should primarily be regarded as an auxological intervention indicated for selected pediatric disorders associated with impaired height prognosis, rather than as a direct treatment for the underlying endocrine condition itself. More details are summarized in Figure 3.

Because maturation and somatic growth are biologically interconnected processes, therapeutic decision-making should adopt an individualized and multidisciplinary approach. Clinical management must consider baseline auxological characteristics, genetic target height, longitudinal growth trajectory, skeletal maturation, pubertal stage, underlying diagnosis, as well as metabolic and cardiometabolic status. Moreover, expectations regarding adult height outcomes should remain clinically realistic and proportional to the quality and consistency of the currently available evidence.

The variability observed in treatment responses further highlights the need for longitudinal follow-up and multidimensional patient assessment. Monitoring strategies should extend beyond linear growth alone and include skeletal maturation, endocrine and metabolic biomarkers, body composition, psychological well-being, and overall quality of life. Future advances in this field will depend on the development of high-quality prospective studies with longer follow-up periods, standardized outcome measures, and greater incorporation of patient-reported outcomes.

In parallel, progress in genetic profiling and biomarker-based precision medicine may contribute to more individualized algorithms and improved prognostic stratification in pediatric endocrine practice.

## 5. Conclusions

GnRHa and rhGH therapies may benefit selected pediatric disorders of pubertal maturation, particularly in terms of pubertal control, auxological outcomes, and skeletal maturation. However, evidence certainty remains limited by the predominance of moderate-risk observational studies, highlighting the need for further randomized and long-term prospective research.

## Figures and Tables

**Table 1:** Quality assessment of randomized controlled trials using the Cochrane Risk of Bias tool version 2 (RoB 2 tool).

Author, Year	D1	D2	D3	D4	D5	Overall
Kariola et al. (2026) [26]	Some concerns	Some concerns	Low	Low	Low	Some concerns
Yang et al. (2019) [40]	Some concerns	Some concerns	Low	Some concerns	Low	Some concerns
Chung et al. (2018) [41]	Some concerns	Some concerns	Low	Low	Low	Some concerns
Kim et al. (2018) [42]	Some concerns	Some concerns	Low	Low	Low	Some concerns
Lee et al. (2016) [44]	Some concerns	Some concerns	Low	Some concerns	Some concerns	Some concerns

Abbreviation: D1: Systematic error originating from the randomization procedure; D2: Systematic error caused by deviations from the planned interventions; D3: Systematic error related to incomplete outcome data; D4: Systematic error in the assessment of outcome; D5: Systematic error in the reporting of results. The overall risk of bias was evaluated using the Cochrane Risk of Bias 2.0 (ROB 2) tool. Judgements were classified as Low risk, Some concerns, or High risk of bias.

**Table 2:** Quality assessment of non-randomized interventional and observational cohort studies using the Risk of Bias in Non-randomized Studies of Interventions (ROBINS-I) tool.

Author, Year	D1	D2	D3	D4	D5	D6	D7	Overall
Akın & Özgen (2025) [27]	Moderate	Low	Low	Low	Moderate	Low	Low	Moderate
Cammisa et al. (2025) [28]	Moderate	Moderate	Low	Low	Moderate	Low	Low	Moderate
Maleki et al. (2025) [30]	Moderate	Moderate	Low	Moderate	Moderate	Low	Low	Moderate
Rabbani et al. (2025) [31]	Moderate	Moderate	Low	Low	Moderate	Low	Low	Moderate
Ünsal et al. (2025) [32]	Moderate	Moderate	Low	Low	Moderate	Low	Low	Moderate
Yu et al. (2024) [33]	Moderate	Moderate	Low	Low	Low	Low	Low	Moderate
Cho et al. (2023) [34]	Moderate	Low	Low	Moderate	Low	Low	Low	Moderate
Shi et al. (2022) [35]	Moderate	Low	Low	Moderate	Low	Low	Low	Moderate
Chung et al. (2021) [36]	Moderate	Moderate	Low	Low	Low	Low	Low	Moderate
Suh et al. (2021) [37]	Moderate	Moderate	Low	Moderate	Moderate	Low	Low	Moderate
Lecka-Ambroziak et al. (2020) [38]	Serious	Moderate	Low	Low	Moderate	Low	Low	Serious
Klein et al. (2020) [39]	Moderate	Moderate	Low	Low	Low	Low	Low	Moderate
Klein et al. (2016) [43]	Serious	Moderate	Low	Low	Low	Low	Low	Moderate
Zenaty et al. (2016) [45]	Moderate	Moderate	Low	Moderate	Low	Low	Moderate	Moderate

Abbreviation: D1: Bias due to confounding; D2: Bias in selection of participants into the study; D3: Bias in classification of exposure; D4: Bias due to departures from intended exposures; D5: Bias due to missing data; D6: Bias in measurement of outcomes; D7: Bias in selection of the reported result. Overall risk of bias was judged as Low, Moderate, Serious, or Critical. For single-arm or post hoc analyses of interventional trials, application of ROBINS-E is limited, and results should be interpreted with caution.

**Table 3:** Quality appraisal of case series using the Joanna Briggs Institute (JBI) Critical Appraisal Checklist for Case Series.

JBI Item	Appraisal Question	Judgment	Comment
1	Were explicit inclusion criteria defined for the case series	Yes	Inclusion criteria for familial CPP with confirmed *MKRN3* mutations were clearly defined.
2	Was the condition assessed consistently and reliability across all participants?	Yes	CPP diagnosis and hormonal evaluation followed standardized clinical and laboratory criteria.
3	Were appropriate and validated methods used to identify the condition?	Yes	Genetic confirmation of *MKRN3* mutations and GnRH stimulation testing were performed.
4	Were consecutive cases included?	Unclear	Consecutive recruitment was not explicitly reported.
5	Was complete inclusion of participants achieved?	Unclear	Small sample size (n = 4) limits certainty regarding complete case capture.
6	Were the participant’s demographic characteristics clearly described?	Yes	Age, sex, family history, and clinical characteristics were reported.
7	Was relevant clinical information of interest clearly reported?	Yes	Detailed auxological, hormonal, and pubertal data were provided.
8	Were outcomes or follow-up findings clearly presented?	Partially	Short- to mid-term outcomes were described; long-term outcomes (e.g., *FAH*) were unavailable.
9	Were the study setting(s) or clinical site(s) clearly specified?	Yes	Patients were managed in a specialized pediatric endocrinology setting.
10	Was statistical analysis appropriate?	Not applicable	Descriptive design without inferential statistical analysis.

Overall risk of bias: High. Limitations related to the small sample size, lack of a comparator group, and unclear consecutive case inclusion restrict internal validity and generalizability. However, the study provides relevant descriptive evidence in a rare form of *MKRN3*-related familial CPP.

## Data Availability

The original contributions presented in this study are included in the article/Supplementary Materials. Further inquiries can be directed to the corresponding authors.

## Supplementary Materials

The following supporting information can be downloaded at: https://www.mdpi.com/article/10.3390/clinpract16050096/s1, Table S1: Search strategy; Table S2: Characteristics of the included studies; Table S3: Summary of clinical outcomes; Table S4: PRISMA 2020 Checklist.

## Abbreviations

The abbreviations used throughout this manuscript are as follows:

ACTH	Adrenocorticotropic hormone
ALT (GPT)	Alanine aminotransferase
AST (GOT)	Aspartate aminotransferase
BA	Bone age
BMI	Body mass index
BP	Bayley–Pinneau
BUN	Blood urea nitrogen
CA	Chronological age
DST	Dexamethasone suppression test
FT4	Free thyroxine
FSH	Follicle-stimulating hormone
GH	Growth hormone
GP	Greulich and Pyle
IGF-1	Insulin-like growth factor 1
IGFBP-3	Insulin-like growth factor binding protein 3
LH	Luteinizing hormone
PAH	Predicted adult height
RBC	Red blood cells
TSH	Thyroid-stimulating hormone
UFC	Urinary free cortisol
WBC	White blood cells
