# Supplementary material for: Effectiveness and Safety of Hormonal Treatments in Children with Growth Disorders: A Systematic Review of Clinical Evidence
Source: Clin Pract. 2026 May 20;16(5):96. doi: 10.3390/clinpract16050096 (PMC13206453; doi:10.3390/clinpract16050096)
Supplement: Supplementary file 1 [file clinpract-16-00096-s001.zip › clinpract-4289777-supplementary.pdf]

Table S1. Search strategy

Date	Database	Search terms	Search strings
7 September 2025 – 18 October 2025	MEDLINE (*PubMed*)	Delayed puberty; precocious puberty; puberty disorders; hormone therapy; GnRH analogues; testosterone; estradiol; hCG; children; adolescents	1) ("Puberty, Delayed"[MeSH] OR "Precocious Puberty"[MeSH]) AND ("Hormone Therapy"[MeSH] OR "Gonadotropin-Releasing Hormone"[MeSH]) AND ("Child"[MeSH] OR "Adolescent"[MeSH])<br>2) ("delayed puberty"[tiab] OR "late puberty"[tiab] OR "precocious puberty"[tiab]) AND ("hormone therapy"[tiab] OR "GnRH agonist"[tiab])<br>3) ("GnRH agonist*" OR "testosterone replacement" OR "estradiol therapy" OR "hCG therapy") AND ("puberty disorder*")
22 September 2025 – 30 November 2025	Embase	Delayed puberty; precocious puberty; hormone therapy; gonadotropin-releasing hormone; agonist; children; adolescent; adolescents; sex steroid; sex steroids; testosterone; estradiol; hCG; chorionic; gonadotropin; puberty disorder; puberty disorders	1) ('delayed puberty'/exp OR 'precocious puberty'/exp) AND ('hormone therapy'/exp OR 'gonadotropin-releasing hormone agonist'/exp) AND ('child'/exp OR 'adolescent'/exp)<br>2) ("delayed puberty" OR "precocious puberty") AND ("sex steroid*" OR testosterone OR estradiol OR "hCG") AND (child* OR adolescen*)<br>3) ('testosterone'/exp OR 'estradiol'/exp OR 'chorionic gonadotropin'/exp) AND ('puberty disorder*')
5 October 2025 – 15 December 2025	CENTRAL (*Cochrane Library*)	Delayed puberty; Precocious puberty; Hormonal therapy; GnRH agonist; GnRH agonists; testosterone therapy; estradiol therapy	1) (Delayed puberty OR Precocious puberty) AND (Hormonal therapy)<br>2) ("GnRH agonist*") AND ("precocious puberty")<br>3) ("testosterone therapy" OR "estradiol therapy") AND ("delayed puberty")
20 October 2025 – 10 January 2026	Scopus	delayed puberty; precocious puberty; hormone therapy; child; children; adolescent; adolescents; GnRH agonist; GnRH agonists pediatric; pediatrics; testosterone replacement; estradiol replacement; puberty disorder; puberty disorders	1) TITLE-ABS-KEY("delayed puberty" OR "precocious puberty") AND ("hormone therapy") AND ("child*" OR "adolescen*")<br>2) ("GnRH agonist*") AND ("precocious puberty") AND (pediatric*)<br>3) ("testosterone replacement" OR "estradiol replacement") AND ("puberty disorder*")
3 November 2025 – 25 January 2026	Web of Science (*WoS*)	precocious puberty; delayed puberty; hormone therapy; GnRH agonist; GnRH agonists; sex steroid therapy; children; adolescent; adolescents; testosterone; estradiol hCG; puberty disorder; puberty disorders	1) TS=("precocious puberty" OR "delayed puberty") AND TS=("hormone therapy")<br>2) TS=("GnRH agonist*" OR "sex steroid therapy") AND TS=("child*" OR "adolescen*")<br>3) TS=("testosterone" OR "estradiol" OR "hCG") AND TS=("puberty disorder*")
12 December 2025 – 31 January 2026	CINAHL (EBSCOhost)	Puberty, Delayed; Precocious Puberty; Hormonal Therapy; delayed puberty; precocious puberty; hormone therapy; GnRH child; children; adolescent; adolescents; testosterone therapy; estradiol therapy; puberty	1) MH "Puberty, Delayed" OR MH "Precocious Puberty" AND MH "Hormonal Therapy"<br>2) ("delayed puberty" OR "precocious puberty") AND ("hormone therapy" OR "GnRH") AND (child* OR adolescen*)<br>3) ("testosterone therapy" OR "estradiol therapy") AND ("puberty")

10 December 2025 – 23 January 2026	LILACS (BVS / DeCS)	puberdade precoce; puberdade tardia; terapia hormonal; Puberdade Precoce; Puberdade Tardia; Terapia Hormonal; GnRH; análogos de GnRH	1) ("puberdade precoce" OR "puberdade tardia") AND ("terapia hormonal")<br>2) (MH:"Puberdade Precoce" OR MH:"Puberdade Tardia") AND MH:"Terapia Hormonal"<br>3) ("GnRH" OR "análogos de GnRH") AND ("puberdade precoce")
10 December 2025 – 23 January 2026	OpenGrey	precocious puberty; delayed puberty; hormonal therapy; hormone treatment; GnRH; gonadotropin-releasing hormone; GnRH analogues; GnRH agonists	1) ("precocious puberty" OR "delayed puberty") AND ("hormonal therapy" OR "hormone treatment")<br>2) ("GnRH" OR "gonadotropin-releasing hormone" OR "GnRH analogues" OR "GnRH agonists") AND ("precocious puberty")
2 May 2026 – 10 May 2026	MEDLINE (*PubMed*)	Delayed puberty; precocious puberty; puberty disorders; hormone therapy; GnRH analogues; testosterone; estradiol; hCG; children; adolescents	1) ("Puberty, Delayed"[MeSH] OR "Precocious Puberty"[MeSH]) AND ("Hormone Therapy"[MeSH] OR "Gonadotropin-Releasing Hormone"[MeSH]) AND ("Child"[MeSH] OR "Adolescent"[MeSH])<br>2) ("delayed puberty"[tiab] OR "late puberty"[tiab] OR "precocious puberty"[tiab]) AND ("hormone therapy"[tiab] OR "GnRH agonist"[tiab])<br>3) ("GnRH agonist*" OR "testosterone replacement" OR "estradiol therapy" OR "hCG therapy") AND ("puberty disorder*")

The literature search was conducted between September 7, 2025 and May 10, 2026 across multiple electronic databases, including MEDLINE (PubMed), Embase, CENTRAL (Cochrane Library), Scopus, Web of Science (WoS), CINAHL Complete (EBSCOhost), LILACS (BVS/DeCS), and OpenGrey. Search strategies combined controlled vocabulary terms (e.g., MeSH, Emtree, DeCS, and database-specific subject headings) with free-text keywords adapted to the syntax and indexing system of each database to maximize sensitivity and comprehensiveness. Core concepts included disorders of pubertal maturation (e.g., delayed puberty, precocious puberty, and puberty disorders), hormone-based therapeutic interventions (e.g., gonadotropin-releasing hormone agonists [GnRHa], testosterone, estradiol, and human chorionic gonadotropin [hCG]), and pediatric populations (children and adolescents). Boolean operators (AND/OR), truncation symbols, title/abstract fields, and controlled indexing terms were applied as appropriate for each platform. An updated search was performed in MEDLINE (PubMed) between May 2 and May 10, 2026 to identify newly published studies and ensure inclusion of the most recent evidence prior to the final synthesis. Abbreviations: GnRH, gonadotropin-releasing hormone; hCG, human chorionic gonadotropin; MeSH, Medical Subject Headings; WoS, Web of Science.

clinics and practice

MDPI

Table S2. Characteristics of the included studies

Ref/ Country	Design	Population / Dx	N	Sex	Age	Inclusion	Intervention	Dose / Regimen	Duration / Follow-up	Comparator	Key results	Safety / AEs	Conclusion
Kariola et al. (2026) [26]<br><br>Finland	RCT, OL	Boys with CDGP	30	30 + controls	– 100% M	– ≥14 yrs	– Delayed puberty<br>– TV 2.5–4 mL; T <5 nmol/L – Tanner G2	– Letrozole<br>– Testosterone	– Lz 2.5 mg/day PO<br>– T 1 mg/kg IM q4w	6m tx + 12m FU	– ↓ Emotionality p=0.04<br>– ↓ Anger/Fear p=0.02<br>– Lz ↑ sociability	No adverse psychosocial effects	Lz/T safe; Lz may improve sociability
Akın & Özgen (2025) [27]<br><br>Turkey	Retrospective obs. cohort	Girls with CPP	117	117 F (100%)	– CA G1 8.59±0.27; G2 7.50±0.47 yrs<br>– BA G1 10.40±0.85; G2 9.39±1.03	– Female<br>– Breast dev. <8 yrs peak<br>– LH >5 IU/L BA≥CA+1<br>– rapid progression<br>– complete data	– GnRHa: leuprolide acetate	– 3.75 mg IM q4w<br>– 7.5 mg if insufficient suppression	– G1 1.97±0.54 yrs<br>– G2 2.91±0.61 yrs<br>– annual FU to FAH	None; baseline reference	– FAH +~4 cm vs PAH p<0.001<br>– no FAH diff.<br>– <8 vs ≥8 yrs<br>– baseline height SDS strongest predictor β=0.572	No osteoporosis; 4/52 BMD Z −1 to −2; Ca/P normal; no severe AEs	GnRHa 7–9 yrs modestly improves FAH; individualize tx; monitor BMI/BH
Cammisa et al. (2025) [28]<br><br>Italy	Single-center retrospective obs. cohort	Girls with idiopathic CPP	34	34 F (100%)	– Diagnosis mean 6.9 yrs; range 1.0–7.9	– Puberty <8 yrs thelarche<br>– GnRH test LH peak >5 IU/L<br>– GnRHa tx; regular FU	– GnRH analogue: triptorelin	– 3.75 mg IM q28d if >20 kg<br>– 1.875 mg q28d if ≤20 kg	– Mean 2.78 yrs in stoppers<br>– range 1.5–4.1<br>– 19/34 stopped	None; baseline reference	– BMI z 0.5 baseline →0.8 at 1 yr (p=0.015) →1.0 1 yr post-stop (p=0.035)<br>– OW/OB prevalence ns	Metabolic impact modest; no major safety signal stated	BMI z increased but obesity prevalence not significantly increased
Chen et al. (2025) [29]<br><br>China	Case series	Familial CPP due to MKRN3 mutation	4	3 F; 1 M	– Onset F 5.7–7.0; M 8.8 yrs	– Advanced BA pubertal LH response<br>– Early gonadal dev.<br>– Positive family history	– GnRHa: triptorelin or leuprorelin	– 3.75 mg monthly	– ≥2 yrs<br>– FU q6m	Within-patient before/after	– FSH ↓ p=0.038<br>– uterine volume ↓ p=0.030<br>– ovarian volume ↓ p=0.007<br>– BAA/PAH ns	No serious AEs; obesity not observed	Safe/effective pubertal suppression; limited effect on BA/height
Maleki et al. (2025) [30]<br><br>Iran	Retrospective longitudinal obs.; up to 10 yrs FU	Children with CPP	196	189 F (96.4%); 7 M (3.6%)	– Signs onset F 6.7±1.4; M 7.9±0.9; tx start F 7.7±1.2; M 9.0±1.0	– Secondary sex signs <8 F/<9 M<br>– progressive CPP<br>– LH>15<br>– LH/FSH>0.66<br>– BA≥CA+1<br>– GnRHa ≥2 yrs	– Depot GnRHa: triptorelin SR (Diphereline)	– 3.75 mg IM q28d >20 kg – 1.87 mg q28d <20 kg<br>– q21d if inadequate	– F 3.4±1.2 yrs<br>– M 3.2±1.0 yrs	None; baseline reference	– Girls FH 161.4±4.2 cm<br>– FH>target p<0.001<br>– BA–CA 1.5→0.7 yrs p<0.001<br>– BMI-SDS ↑ p=0.033	Generally tolerated; no major limiting AEs	Improves FH esp. girls; BMI-SDS may increase; monitor metabolism
Rabbani et al. (2025) [31]<br><br>Iran	Phase IV registry; real-world obs.	Children/adolescents on GH; mainly GHD	20465	12,457 F (60.9%); 8,008 M (39.1%)	– Mean 10.56±2.90 yrs	– 2–18 yrs<br>– GH-naïve pediatric endocrinologist indication<br>– Orchid-Life registry<br>– Continuous Cinnatropin	– Biosimilar rhGH: Cinnatropin	– Mean ~0.034 mg/kg/day SC daily	– 12 months<br>– longitudinal complete n=405	None; baseline reference	– HSDS longitudinal −1.84→−1.49<br>– gain ~+0.4 SDS<br>– older age inversely correlated with gain<br>– >80% prepubertal achieved target HV	≥1 AE 3.57%; mostly mild; SAE 0.08%; 2 deaths unrelated	Clinically meaningful height improvement, better when started earlier; mostly mild AEs

Ünsal et al. (2025) [32]<br><br>Turkey	Retrospective longitudinal cohort	Boys with idiopathic CPP	80	80 M (100%)	– Onset 8.2±2.2; diagnosis/tx ~9.0±2.2 yrs	– Testicular vol >3 mL<br>– Accelerated growth BA≥CA+1<br>– Pubertal LH<br>– Normal MRI	– GnRHa monotherapy: leuprolide acetate	– 3.75 mg q4w	– Mean 2.8±0.5 yrs<br>– FH 7.0±1.3 yrs post-tx<br>– FH n=58	None; baseline reference	– Overall BMI-SDS ns during tx<br>– FH BMI-SDS lower vs pre $p<0.001$<br>– OB subgroup BMI-SDS ↓ $p<0.001$<br>– LH/testosterone suppressed	No major safety issues stated	BMI response depends baseline BMI; lifestyle counseling recommended
Yu et al. (2024) [33]<br><br>China	Phase III open-label single-arm	Children with CPP	66	93.9% F; 6.1% M	– Girls <9 yr; boys <10 yr	– Tanner ≥II<br>– LH peak ≥6 IU/L<br>– BA–CA >1 yr	– Triptorelin pamoate	– 22.5 mg IM at day 1 and month 6	12 months	None	– LH suppression 100% at 6m, 98.5% at 12m<br>– GV 9.82→5.17<br>– BA/CA 1.27→1.21<br>– ≥93% Tanner stable	Drug-related TEAEs 19.7%; no grade ≥3; no discontinuations; weight gain common	Effective and safe sustained HPG-axis suppression
Cho et al. (2023) [34]<br><br>South Korea	Retrospective longitudinal obs.	Girls with idiopathic CPP	210	210 F (100%)	– Tx start ~8.2–8.3 yrs	– Advanced BA<br>– pubertal LH response<br>– CPP criteria	– GnRHa ± GH	– Leuprolide q28d<br>– GH when PAH low/GV declined	– GnRHa ~3 yrs – GH ~3 yrs	GnRHa vs GnRHa+GH	– FAH ns: 161.07±4.78 vs 159.63±3.86 cm<br>– height gain greater combo 9.22 vs 4.72 cm $p<0.001$<br>– BMI-SDS ns	No significant tx-related AEs; no metabolic/thyroid/glucose abnormalities	Combo improves height gain but not FAH; GH individualized
Shi et al. (2022) [35]<br><br>China (Wuhan)	Single-center retrospective obs. cohort	Girls with idiopathic CPP	80	80 F (100%)	– CA G1 8.02±0.83; G2 8.73±0.94;<br>– BA G1 9.24±1.07; G2 10.47±1.01	– ICPP; min tx ≥30 months<br>– FU q3m<br>– BA q6m	– GnRHa alone vs GnRHa+rhGH	– GnRHa 3.75 mg q4w<br>– rhGH 0.05–0.066 mg/kg/day SC	– ≥30 months	GnRHa alone (n=34) vs combo (n=46)	– Combo higher height at 12/24/30m<br>– GV yr1 8.74 vs 6.18 $p<0.0001$<br>– PAH gain 8.2 vs 2.2 cm<br>– BMI ns	No serious AEs; no discontinuations; no abnormal BA acceleration	Combo improves growth/PAH in poor prognosis without added metabolic/skeletal risk
Chung et al. (2021) [36]<br><br>South Korea	Retrospective case-control obs.	Girls with CPP vs healthy controls	32	32 F (100%)	– Overall 8.6±0.7 yrs; CPP 8.7±0.5; control 8.3±0.9	– CPP: breast dev <8 BA advanced >1<br>– peak LH >5<br>– ophth FU ≥1 yr	– CPP group: leuprolide acetate depot	– 3.75 mg SC q28d	– ≥12 months	Healthy controls	– SE change CPP −0.64 vs control −0.85 D ns - AL change 0.35 vs 0.55 mm ns<br>– AL change linked to height gain $\beta=0.691$ $p=0.039$	No tx-related AEs reported	Myopia progression comparable to controls; linear growth may drive AL elongation
Suh et al. (2021) [37]<br><br>South Korea (Seoul)	Retrospective obs. cohort	Children with HH; CPP subgroup	30	16 F (53.3%); 14 M (46.7%)	– HH dx 4.2±2.9; CPP dx 5.7±3.1 yrs	– CPP: peak LH ≥5<br>– puberty <8 F/<9 M<br>– HH-associated	– GnRH agonist: leuprolide or triptorelin	– 3.75 mg q4w	– 3.1±2.2 yrs mean	None; baseline reference	– CPP in 24/30 BA–CA 3.3→2.0 yrs $p=0.002$<br>– PAH improved esp. girls	GnRHa well tolerated	GnRHa safe/effective in HH-related CPP; improves skeletal maturation/height prognosis
Lecka-Ambroziak et al. (2020) [38]	Retrospective obs. cohort	Children with PWS	49	PA: 8/11 F; NA: 9 M/5 F	– End FU 12.1±6.8 yrs; rhGH start PA 4.5±3.2; NA 7.1±2.9	– PWS treated with rhGH	– rhGH	– ~0.025 mg/kg/day SC	– PA 10.2±2.7 yrs	PA vs normal adrenarche	– PA 22.5%<br>– central puberty onset ns; FH SDS ns	rhGH tolerated; no AE increase in	PA common in PWS on rhGH but does not

Poland		on rhGH; PA vs NA				– PA vs normal adrenarche analysis			– NA 8.5±3.1 yrs		– BA/CA ~1<br>– metabolic profile comparable	PA; no discontinuations	affect puberty, growth response, or safety
Klein et al. (2020) [39]<br><br>United States	Phase III multicenter open-label single-arm	Children with CPP	64	60/62 F (96.8%); 2/62 M	– Mean 7.5±0.1 yrs	– Girls 2–8; boys 2–9<br>– Tanner≥2<br>– BA≥CA+1; peak LH><br>– no prior GnRHa	– Leuprolide acetate 45 mg 6-month depot	– 45 mg SC; baseline + week 24	– 48 weeks	None	– LH suppression 87% wk24; ~86% wk48<br>– GV 8.9→6.0<br>– BA–CA 3.0→2.7 *p*<0.001<br>– Tanner stable/regressed 97%	No serious tx-related AEs; injection site pain 31%; no discontinuations	6-month SC leuprolide effective, safe, convenient
Yang et al. (2019) [40]<br><br>Republic of Korea	Phase III randomized active-controlled open-label	Infants/toddlers with genetic PWS	34	Eutropin 11F/5M; Genotropin 7F/6M	– 2.3–24 months; Eutropin 4.81±2.04 mo; comparator 8.04±5.81 mo	– Genetic PWS prepubertal<br>– GH-naïve or limited prior rhGH<br>– no other growth cause	– rhGH: Eutropin vs Genotropin	– Target 0.24 mg/kg/wk SC 6x/wk; titrated from 0.084	– 52 weeks + 4-week safety FU	Active comparator Genotropin	– Height SDS +0.75 vs +0.95<br>– LBM ↑fat% ↓<br>– motor/cognitive scores ↑ both, no group diff major	AEs common; SAEs 47.1% vs 41.2%; sleep apnea 1 case; discontinuation 5.9% vs 17.6%	rhGH improves growth, body composition, neurodevelopment; comparable safety
Chung et al. (2018) [41]<br><br>South Korea	Phase III randomized multicenter open-label delayed-control	Prepubertal children with ISS	90	Tx 30M/29F; Control 17M/13F	– Tx 6.79±1.54; control 6.83±1.61 yrs	– Height <3rd percentile prepubertal<br>– normal GH tests<br>– BA limits; BA–CA≤3 yrs	– rhGH Saizen	– 0.067 mg/kg/day SC 6d/wk	– 12 months tx – Delayed control first 6m none then 6m rhGH	Delayed-treatment control	– HV 6m 10.08 vs 5.92 cm/yr; diff +3.47 *p*<0.0001<br>– height SDS +0.51 vs control *p*<0.0001<br>– BMI ns	AEs mostly mild; rare unrelated SAEs; no deaths/discontinuations	rhGH improves short-term HV with favorable safety; adult height unknown
Kim et al. (2018) [42]<br><br>South Korea	Phase III randomized open-label multicenter controlled	Prepubertal children with ISS	70	39 M (55.7%); 31 F (44.3%)	– Age 4.0–12.1; mean 6.96±2.07; BA 5.32±2.24	– Height SDS 2.37±0.50<br>– normal GH stimulation<br>– Tanner I	– rhGH Growtropin-II	– 1.11 IU/kg/wk (~0.37 mg/kg/wk), SC 6–7 doses/wk	– 52 weeks control observation<br>– 26w then rhGH 26w	Delayed-treatment control	– Week26 HV 10.68 vs 5.72 *p*<0.001<br>– height SDS +0.63 vs +0.06 *p*<0.001<br>– BMI ns; BA appropriate	Mostly mild infections; rare unrelated SAEs; one mild rash; no discontinuations	Improves HV and height SDS without relevant BA acceleration
Klein et al. (2016) [43]<br><br>USA, Chile, Mexico	Phase III open-label non-comparative multicenter	Treatment-naïve children with CPP	44	39 F (88.6%); 5 M (11.4%)	– Overall 7.41±1.28; girls 7.21±1.22; boys 9.0	– Puberty signs <8 F/<9 M<br>– Tanner≥2<br>– BA≥CA+1<br>– LH ≥6 after stimulation<br>– tx ≤18m onset	– Triptorelin embonate 6-month depot	– 22.5 mg IM at months 0 and 6	– 12 months; visits months 1,2,3,6,9,12	None	– LH suppression 93.2% at m6; 97.7% at m12<br>– BA/CA 1.40→1.34<br>– Tanner stable/regressed ~89–91%	AEs 75%, 89% mild; related AEs 9.1%; no discontinuations	6-month triptorelin highly effective/safe; may improve adherence
Lee et al. (2016) [44]<br><br>United States	Post hoc randomized open-label phase III; dose analysis	Previously treated CPP transitioning	42	Predominantly F; few M	– Girls 2–10; boys 2–11; mean ~8–9 yrs	– CPP onset <8 F/<9 M<br>– BA≥CA+1<br>– prior GnRHa ≥6m<br>– peak LH<4 at entry<br>– CNS pathology excluded	– 3-month leuprolide acetate depot	– 11.25 mg vs 30 mg IM q3m; baseline + wk12	– Core 6m transition 12w<br>– Pooled extension 36m for basal LH	Dose comparison	– Suppression: 81% with 11.25 mg vs 100% with 30 mg<br>– basal LH <0.6 IU/L sensitivity 80%, specificity 70%	AEs higher in 30 mg; no new safety signals; no discontinuations	3-month leuprolide maintains suppression, esp 30 mg;

		formulation											basal LH useful monitor
Zenaty et al. (2016) [45]<br><br>France, Belgium, Italy, Spain	Phase III multicenter open-label non-comparative; retrospective formulation comparison	Children with CPP	101	Predominantly F (>90%)	– Not detailed	– Breast dev <8 F testicular enlargement<br>– <9 M<br>– BA–CA>1<br>– peak LH >5 or ≥7	– Triptorelin pamoate vs acetate	– Pamoate 11.25 mg IM q3m; acetate 11.25 mg	– 6 months primary endpoint	Historical triptorelin acetate	– LH suppression m3 83.8% vs 82.8%<br>– m6 86.5% vs 96.8%<br>– pubertal progression slowed	AEs mild–moderate; no toxicity discontinuations	Pamoate q3m effective and safe, comparable to acetate

The table summarizes the main methodological and clinical characteristics of the studies included in the qualitative synthesis, including reference and country, study design, diagnostic population, sample size, sex distribution, age, eligibility criteria, intervention type, therapeutic regimen and dosage, treatment duration and follow-up, comparator group, principal effectiveness outcomes, safety findings, and overall conclusions. Studies encompassed randomized controlled trials (RCTs), phase III clinical trials, retrospective and prospective observational cohorts, case–control studies, case series, and real-world registry-based investigations. Interventions included gonadotropin-releasing hormone agonists (GnRHa), recombinant human growth hormone (rhGH), testosterone, estradiol, letrozole, and human chorionic gonadotropin (hCG), administered for conditions such as central precocious puberty (CPP), constitutional delay of growth and puberty (CDGP), idiopathic short stature (ISS), Prader–Willi syndrome (PWS), hypothalamic hamartoma (HH)-associated CPP, and familial CPP related to *MKRN3* mutations. Main outcomes included pubertal suppression, growth velocity (GV), height standard deviation score (height SDS/HSDS), predicted adult height (PAH), final adult height (FAH), bone age (BA) progression, body mass index (BMI/BMI-SDS), endocrine response, psychosocial variables, and treatment-related adverse events (AEs). Abbreviations: BA, bone age; BAA, bone age advancement; BMI, body mass index; BMI-SDS, body mass index standard deviation score; CA, chronological age; CDGP, constitutional delay of growth and puberty; CPP, central precocious puberty; Dx, diagnosis; FAH, final adult height; FU, follow-up; F, female; GH, growth hormone; GnRHa, gonadotropin-releasing hormone agonist; GV, growth velocity; hCG, human chorionic gonadotropin; HH, hypothalamic hamartoma; HPG, hypothalamic–pituitary–gonadal; HSDS, height standard deviation score; IM, intramuscular; ISS, idiopathic short stature; LH, luteinizing hormone; Lz, letrozole; M, male; MRI, magnetic resonance imaging; ns, non-significant; obs., observational; OL, open-label; PA, premature adrenarche; PAH, predicted adult height; PO, oral administration; PWS, Prader–Willi syndrome; q4w, every 4 weeks; q6m, every 6 months; RCT, randomized controlled trial; rhGH, recombinant human growth hormone; SAE, serious adverse event; SC, subcutaneous; SDS, standard deviation score; SR, sustained release; T, testosterone; Tanner G2, Tanner genital stage II; TEAEs, treatment-emergent adverse events; TV, testicular volume; tx, treatment; yrs, years.

Table S3. Summary of clinical outcomes

Domain	Population	Intervention	Main Findings
**Growth Outcomes and Final Adult Height**	*Girls with idiopathic CPP* [27,30]	GnRHa	• FAH gain vs. PAH: Δ ≈ +4.0 cm (p < 0.001).<br>• FAH: 161.4 ± 4.2 cm [30].<br>• BA–CA: 1.5 ± 1.3 → 0.7 ± 1.1 years (Δ −0.8; p < 0.001).<br>• Effect of age at start: no significant difference (<8 vs. ≥8 years; p > 0.05) [27]
	Girls with idiopathic CPP [34,35]	GnRHa + rhGH vs. GnRHa	• FAH–PAH gain: 9.22 ± 6.03 vs. 4.72 ± 5.01 cm (Δ +4.5 cm; *p* < 0.001) [34].<br>• FAH: 159.63 ± 3.86 vs. 161.07 ± 4.78 cm (*p* = 0.174).<br>• PAH gain: +8.2 ± 4.9 vs. +2.2 ± 6.4 cm [35].
	Boys with idiopathic CPP [32]	GnRHa	• Height SDS: 1.1 ± 1.3 → −0.7 ± 1.3 (Δ −1.8; *p* < 0.01).<br>• Final height: within normal range.
	Children with GHD [31]	rhGH	• HSDS: −1.84 ± 1.18 → −1.49 ± 1.13 (Δ +0.35 SDS).<br>• Mean HSDS gain: ≈ +0.4 SDS (12 months).<br>• Age effect: r = −0.11 to −0.12 (*p* < 0.001).
	ISS [41,42]	rhGH	• Height velocity: 5.63 ± 1.62 → 10.08 ± 1.92 cm/year (Δ +4.45; p < 0.0001) [41].<br>• Between-group ΔHV: +3.47 cm/year (95% CI 2.17–4.78; *p* < 0.0001).<br>• Height SDS: Δ +0.5–0.6 SDS.
	PWS [38,40]	rhGH	• Height SDS: Δ +0.75 ± 0.59 to +0.95 ± 0.66 (*p* < 0.001) [40].<br>• Final height SDS: −1.1 ± 1.5 vs. −1.5 ± 1.0 (*p* = 0.40) [38].
	Familial CPP (MKRN3) [29]	GnRHa	• Height SDS: slight decrease (ns).<br>• PAH: no significant improvement (*p* > 0.05).
**Skeletal Maturation and Bone Age Advancement**	*CPP* [27,30,33,43]	GnRHa	• BA–CA: 1.5 ± 1.3 → 0.7 ± 1.1 years (Δ −0.8; *p* < 0.001) [30].<br>• BA/CA: 1.27 → 1.21 (Δ −0.06) [33].<br>• Stabilization: >90% of patients across studies.
	CPP [37]	GnRHa	• BA–CA: 3.3 → 2.0 years (Δ −1.3; *p* = 0.002).<br>• Height SDS (BA-adjusted): increased.
	CPP secondary to hypothalamic hamartoma [35]	GnRHa	• BA–CA: ↓ 3.3 → 2.0 years (*p* = 0.002)<br>• Height SDS for BA ↑
	ISS & PWS [38,40–42]	rhGH	• BA/CA: ≈1.0 (no abnormal acceleration).<br>• BA progression: proportional to CA.
	Familial CPP (MKRN3) [29]	GnRHa	• ΔBA–CA: <0.3 years (ns).
**BMI and Anthropometric Composition**	*Girls with idiopathic CPP* [27,28,30]	GnRHa	• BMI-SDS: 1.2 ± 1.0 → 1.3 ± 0.9 (Δ +0.1; *p* = 0.033) [30].<br>• BMI z-score: 0.5 → 0.8 (Δ +0.3; *p* = 0.015) [28].<br>• Post-treatment: normalization or ↓ below baseline (*p* = 0.002) [27].<br>• Obesity prevalence: no significant change (*p* = 0.289).
	Boys with CPP	GnRHa	• BMI-SDS: 1.3 ± 0.9 → 1.5 ± 0.7 → 0.9 ± 1.5 (final; *p* < 0.001).

	[32]		• OB subgroup: Δ −0.6 SDS (*p* < 0.001).
	ISS [41,42]	rhGH	• BMI-SDS: no significant change (*p* > 0.05).
	PWS [38,40]	rhGH	• Lean mass: +2377.8 ± 536.3 g (p < 0.001).<br>• Fat mass: −8.1 ± 9.9% (p ≤ 0.005).
	Familial CPP (MKRN3) [29]	GnRHa	4) BMI-SDS: decreased.<br>5) Obesity prevalence: reduced.
**Endocrine, Hormonal and Psychocognitive Outcomes**	*CPP* [29,32,33,39,43]	GnRHa	• LH suppression: 93–100% at 6–12 months.<br>• Example: 41/44 (93.2%) at 6 months; 97.7% at 12 months [43].<br>• Basal LH: 1.7 ± 1.1 → 0.3 ± 0.2 mIU/mL [32].
	CDGP boys [26]	Letrozole vs. testosterone	• Emotionality: ↓ (*p* = 0.04).<br>• Anger/fear: ↓ (*p* = 0.02).<br>• Sociability (Lz vs T): *p* = 0.019 (6m); *p* = 0.001 (12m).
	GHD/ISS/PWS [31,40–42]	rhGH	• IGF-1 SDS: Δ +1.0 to +2.0 SDS.<br>• IGFBP-3: increased.<br>• Target HV achieved: >80% [31].
	PWS [40]	rhGH	• Motor score: +40.4 ± 7.8 (*p* < 0.001).<br>• Cognitive score: +56.8 ± 14.6 (*p* < 0.001).
**Safety Outcomes**	*All populations* [26–45]	GnRHa / rhGH / others	• AEs: mostly mild (≈75% incidence in some cohorts) [43].<br>• Drug-related AEs: ~9–20%.<br>• SAEs: rare (<1%).<br>• Discontinuation: <1–2%.
	Long-term follow-up [27,30,32,38]	GnRHa / rhGH	• Bone health: no osteoporosis reported.<br>• BMD Z-score: −1 to −2 in 4/52 cases [27].<br>• Metabolic profile: stable (glucose/lipids).

The table synthesizes the principal findings of the studies included in the qualitative review according to clinical domain, study population, therapeutic intervention, and main outcomes. Effectiveness domains included auxological outcomes and final adult height (FAH), skeletal maturation and bone age (BA) progression, body mass index (BMI) and anthropometric composition, endocrine and hormonal responses, psychocognitive effects, and treatment safety. Interventions primarily involved gonadotropin-releasing hormone agonists (GnRHa), recombinant human growth hormone (rhGH), testosterone, and letrozole in populations with central precocious puberty (CPP), constitutional delay of growth and puberty (CDGP), growth hormone deficiency (GHD), idiopathic short stature (ISS), Prader–Willi syndrome (PWS), and familial CPP associated with MKRN3 mutations. Main effectiveness outcomes included changes in predicted adult height (PAH), FAH, height standard deviation score (height SDS/HSDS), height velocity (HV), BA advancement, hormonal suppression of the hypothalamic–pituitary–gonadal (HPG) axis, metabolic and body composition changes, and psychosocial development. Safety outcomes comprised adverse events (AEs), serious adverse events (SAEs), treatment discontinuation, and long-term endocrine, skeletal, and metabolic monitoring. Abbreviations: AE, adverse event; AMH, anti-Müllerian hormone; BA, bone age; BA–CA, difference between bone age and chronological age; BA/CA, bone age-to-chronological age ratio; BMI, body mass index; CDGP, constitutional delay of growth and puberty; CPP, central precocious puberty; FAH, final adult height; FSH, follicle-stimulating hormone; GH, growth hormone; GHD, growth hormone deficiency; GnRHa, gonadotropin-releasing hormone agonist; HPG axis, hypothalamic–pituitary–gonadal axis; HSDS, height standard deviation score; HV, height velocity; IGF-1, insulin-like growth factor 1; IGFBP-3, insulin-like growth factor-binding protein 3; ISS, idiopathic short stature; LH, luteinizing hormone; MKRN3, makorin ring finger protein 3; ns, non-significant; OB, obese; OW, overweight; PAH, predicted adult height; PWS, Prader–Willi syndrome; rhGH, recombinant human growth hormone; SAE, serious adverse event; SDS, standard deviation score; SGA, small for gestational age. Symbols: ↑ indicates increase, ↓ indicates decrease, and = indicates no statistically significant difference.

Table S4. PRISMA 2020 Checklist

Section and Topic	Item #	Checklist item	Location where item is reported
**TITLE**			
Title	1	Identify the report as a systematic review.	**Line 1-4:** "[…] Effectiveness and Safety of Hormonal Treatments in Children with Growth Disorders: A Systematic Review of Clinical Evidence […]"
**ABSTRACT**			
Abstract	2	See the PRISMA 2020 for Abstracts checklist.	**Lines 15-34 Abstract**: Abstract: Background, Methods, Results, Conclusions. Includes databases, dates, PROSPERO registration, number of included studies, main outcomes, safety, limitations and conclusions.
**INTRODUCTION**			
Rationale	3	Describe the rationale for the review in the context of existing knowledge.	**Lines 43-112 ntroduction**: *Introduction: "Growth disorders, including central precocious puberty and delayed puberty…" and "heterogeneity in clinical practice… underscores the necessity for a rigorous and integrative synthesis."*
Objectives	4	Provide an explicit statement of the objective(s) or question(s) the review addresses.	**Lines 100-112 End of Introduction**: *End of Introduction: "the goal of this review is to critically analyze the effectiveness and safety profile of hormone therapy in pediatric pubertal disorders."*
**METHODS**			
Eligibility criteria	5	Specify the inclusion and exclusion criteria for the review and how studies were grouped for the syntheses.	**Section 2.2**: *Section 2.2. Study Selection: criteria by date, design, age, diagnosis, intervention, language, full-text availability and outcomes.*
Information sources	6	Specify all databases, registers, websites, organisations, reference lists and other sources searched or consulted to identify studies. Specify the date when each source was last searched or consulted.	**Section 2.1**: *Section 2.1: MEDLINE, EMBASE, CENTRAL, Scopus, Web of Science, CINAHL, LILACS and OpenGrey; search conducted from 1 September 2025 to 31 March 2026.*
Search strategy	7	Present the full search strategies for all databases, registers and websites, including any filters and limits used.	**Section 2.1 and Supplementary Table S1**. Includes MeSH/free-text terms and MEDLINE core query.
Selection process	8	Specify the methods used to decide whether a study met the inclusion criteria of the review, including how many reviewers screened each record and each report retrieved, whether they worked independently, and if applicable, details of automation tools used in the process.	**Section 2.1 and 2.2:** two reviewers independently screened titles/abstracts and full texts; discrepancies resolved by consensus.
Data collection process	9	Specify the methods used to collect data from reports, including how many reviewers collected data from each report, whether they worked independently, any processes for obtaining or confirming data from study investigators, and if applicable, details of automation tools used in the process.	**Section 2.3:** two authors independently extracted data using a standardized PICO-based template; pilot extraction following Cochrane Handbook v6.4.
Data items	10a	List and define all outcomes for which data were sought. Specify whether all results that were compatible with each outcome domain in each study were sought (e.g. for all measures, time points, analyses), and if not, the methods used to decide which results to collect.	**Section 2.3**: pubertal progression, growth parameters, BA, hormonal/metabolic changes, psychological well-being, adverse events, effect measures and follow-up.

Section and Topic	Item #	Checklist item	Location where item is reported
	10b	List and define all other variables for which data were sought (e.g. participant and intervention characteristics, funding sources). Describe any assumptions made about any missing or unclear information.	**Section 2.3:** study characteristics, design, diagnostic criteria, sample size, age, sex, Tanner stage, hormonal profile, intervention type, dosage, duration and comparator details. Funding sources are not clearly reported as extracted variables.
Study risk of bias assessment	11	Specify the methods used to assess risk of bias in the included studies, including details of the tool(s) used, how many reviewers assessed each study and whether they worked independently, and if applicable, details of automation tools used in the process.	**Section 2.4:** RoB 2 for RCTs, ROBINS-I for non-randomized/observational studies, JBI checklist for case series; two reviewers assessed independently.
Effect measures	12	Specify for each outcome the effect measure(s) (e.g. risk ratio, mean difference) used in the synthesis or presentation of results.	**2.3. Data extraction** No meta-analysis was performed due to the methodological heterogeneity among the included studies regarding study design, outcome measures, interventions, and follow-up durations. Therefore, the review adopted a qualitative synthesis approach. Data extraction included statistical findings and effect measures reported by the original studies, such as mean differences, p-values, beta coefficients, confidence intervals, and follow-up duration when available.
Synthesis methods	13a	Describe the processes used to decide which studies were eligible for each synthesis (e.g. tabulating the study intervention characteristics and comparing against the planned groups for each synthesis (item #5)).	**Section 2.2 and 3.2***:* studies grouped by diagnosis, intervention and outcome domains in qualitative synthesis.
	13b	Describe any methods required to prepare the data for presentation or synthesis, such as handling of missing summary statistics, or data conversions.	**Section 2.3:** *standardized PICO extraction template and pilot extraction following Cochrane Handbook.*
	13c	Describe any methods used to tabulate or visually display results of individual studies and syntheses.	**Section 3.2 and Tables S2**: Section 3.2, Tables 1–3, Supplementary Tables S1–S3, Figures 1–3.
	13d	Describe any methods used to synthesize results and provide a rationale for the choice(s). If meta-analysis was performed, describe the model(s), method(s) to identify the presence and extent of statistical heterogeneity, and software package(s) used.	**Section 3.4 and Discussion**: qualitative synthesis performed; meta-analysis not conducted due to substantial clinical and methodological heterogeneity.
	13e	Describe any methods used to explore possible causes of heterogeneity among study results (e.g. subgroup analysis, meta-regression).	**Partially reported in Section 4.4**: heterogeneity discussed by design, population, intervention, outcomes and follow-up. No formal subgroup/meta-regression analysis reported.
	13f	Describe any sensitivity analyses conducted to assess robustness of the synthesized results.	Not reported. No sensitivity analyses were conducted because quantitative synthesis was not performed.
Reporting bias assessment	14	Describe any methods used to assess risk of bias due to missing results in a synthesis (arising from reporting biases).	**Section 2.4:** Partially reported through RoB 2 and ROBINS-I domains on selective reporting. No funnel plot or formal publication-bias assessment reported.
Certainty assessment	15	Describe any methods used to assess certainty (or confidence) in the body of evidence for an outcome.	Not reported. No GRADE or equivalent certainty assessment described.
**RESULTS**			
Study selection	16a	Describe the results of the search and selection process, from the number of records identified in the search to the number of studies included in	**Section 3.1 and Figure 1**: 162 records identified, 29 duplicates removed, 133 screened, 46 full texts assessed, 26 excluded, 20 studies included.

Section and Topic	Item #	Checklist item	Location where item is reported
		the review, ideally using a flow diagram.	
	16b	Cite studies that might appear to meet the inclusion criteria, but which were excluded, and explain why they were excluded.	**Section 3.1. and Figure 1**: Section 3.1 reports exclusion reasons but does not cite individual excluded studies.
Study characteristics	17	Cite each included study and present its characteristics.	**Section 3.2 and Supplementary Table S2**: 20 studies [26–45], 21,737 children, designs, populations, interventions and outcomes described.
Risk of bias in studies	18	Present assessments of risk of bias for each included study.	**Section 3.3 and Tables 1–3:** RoB 2, ROBINS-I and JBI assessments.
Results of individual studies	19	For all outcomes, present, for each study: (a) summary statistics for each group (where appropriate) and (b) an effect estimate and its precision (e.g. confidence/credible interval), ideally using structured tables or plots.	**Section 3.4:** growth, BA, BMI, endocrine, psychocognitive and safety outcomes with statistics where available.
Results of syntheses	20a	For each synthesis, briefly summarise the characteristics and risk of bias among contributing studies.	**Sections 3.3 and Sections 3.4 "**Sections 3.3, 3.4 and Discussion.
	20b	Present results of all statistical syntheses conducted. If meta-analysis was done, present for each the summary estimate and its precision (e.g. confidence/credible interval) and measures of statistical heterogeneity. If comparing groups, describe the direction of the effect.	No meta-analysis conducted. Narrative synthesis reported due to heterogeneity.
	20c	Present results of all investigations of possible causes of heterogeneity among study results.	**Section 4.4:** heterogeneity discussed narratively; no formal statistical exploration.
	20d	Present results of all sensitivity analyses conducted to assess the robustness of the synthesized results.	Not applicable; no sensitivity analyses conducted.
Reporting biases	21	Present assessments of risk of bias due to missing results (arising from reporting biases) for each synthesis assessed.	Partially reported in risk-of-bias tables; no formal synthesis-level reporting bias assessment.
Certainty of evidence	22	Present assessments of certainty (or confidence) in the body of evidence for each outcome assessed.	Not reported; no GRADE assessment included.
**DISCUSSION**			
Discussion	23a	Provide a general interpretation of the results in the context of other evidence.	**Section 4:** interpretation of GnRHa and rhGH effects in context of recent evidence.
	23b	Discuss any limitations of the evidence included in the review.	**Section 4.5:** heterogeneity, observational designs, surrogate outcomes, limited long-term follow-up.
	23c	Discuss any limitations of the review processes used.	**Section 4.4:** absence of meta-analysis, heterogeneity, limited comparability and inconsistent psychosocial outcomes.
	23d	Discuss implications of the results for practice, policy, and future research.	**Section 4.5 and Figure 3**: individualized therapy, specialist supervision, longitudinal monitoring, future prospective studies.
**OTHER INFORMATION**			
Registration and protocol	24a	Provide registration information for the review, including register name and registration number, or state that the review was not registered.	**Section 2.1**: " […]The study protocol was prospectively registered in the PROSPERO database (CRD420251068048) (https://www.crd.york.ac.uk/PROSPERO/view/CRD420251068048), thereby ensuring methodological transparency and adherence to internationally recognized standards for systematic reviews. […]"

Section and Topic	Item #	Checklist item	Location where item is reported
	24b	Indicate where the review protocol can be accessed, or state that a protocol was not prepared.	**Section 2.1**: " […] The study protocol was prospectively registered in the PROSPERO database (CRD420251068048) (https://www.crd.york.ac.uk/PROSPERO/view/CRD420251068048), thereby ensuring methodological transparency and adherence to internationally recognized standards for systematic reviews."[…]"
	24c	Describe and explain any amendments to information provided at registration or in the protocol.	No amendments or deviations from protocol are described.
Support	25	Describe sources of financial or non-financial support for the review, and the role of the funders or sponsors in the review.	**Funding:** " […]This research received no external funding […]".
Competing interests	26	Declare any competing interests of review authors.	**Conflicts of Interest:** " […] The authors declare no conflicts of interest. The funders had no role in the design of the study; in the collection, analysis, or interpretation of data; in the writing of the manuscript; or in the decision to publish the results […]"
Availability of data, code and other materials	27	Report which of the following are publicly available and where they can be found: template data collection forms; data extracted from included studies; data used for all analyses; analytic code; any other materials used in the review.	**Data Availability Statement:** "The data supporting the findings of this study are available within the article and its Supplementary Materials".

From: Page MJ, McKenzie JE, Bossuyt PM, Boutron I, Hoffmann TC, Mulrow CD, et al. The PRISMA 2020 statement: an updated guideline for reporting systematic reviews. BMJ 2021;372:n71. doi: 10.1136/bmj.n71

For more information, visit: http://www.prisma-statement.org/
